# CREAT: A CRISPR‐Based Genome Trimming Strategy for Systematic Identification of Dispensable Regions and Rapid Genome Reduction

**DOI:** 10.1002/advs.76042

**Published:** 2026-06-29

**Authors:** Guanhua Yuan, Zhe Gao, Yingxuan Qi, Yang Zhang, Xuhui Tian, Pengpeng Zhao, Xu Feng, Qunxin She

**Affiliations:** ^1^ CRISPR and Archaea Biology Research Center State Key Laboratory of Microbial Technology Shandong University Qingdao China; ^2^ State Key Laboratory of Microbial Metabolism School of Life Sciences and Biotechnology Shanghai Jiao Tong University 800 Dongchuan Road Minhang District Shanghai China

**Keywords:** classification of essential vs. nonessential regions, CRISPR‐Cas, genome reduction, multi‐homology arms, synthetic genomic fragments, λ‐Red

## Abstract

The construction of minimal‐genome microbes offers an ideal platform for understanding fundamental biological processes and synthetic biology, yet the research is hindered by incomplete lists of essential genes in microbes and by multiple rounds of genome trimming with a trial‐and‐error nature. To address this, we introduce CREAT (CRISPR‐based genome trimming with a multi‐homology‐arm template)—a streamlined approach that integrates CRISPR‐targeted genome cleavage and homology arm walking to classify essential from non‐essential genomic subregions, thus providing the basis for predicting essential genes in a given organism. These essential genes were then assembled into synthetic gene cassettes for one‐step replacement of the targeted non‐deletable genomic regions for further genome trimming. Eight consecutive rounds of CREAT genome trimming achieved a 20.8% reduction in genome size in *Saccharolobus islandicus*. Furthermore, Cas9‐based CREAT genome trimming was developed for *Bacillus subtilis* and *Escherichia coli*, with efficiency greatly enhanced by the λ‐Red recombinase in the latter. Together, this iterative application of CREAT provides a scalable and generally applicable strategy for rapidly constructing minimal genomes across diverse microorganisms.

## Graphical abstract

Current genome‐reduction methods work in a trial‐and‐error fashion, constituting a long‐standing bottleneck in the research. We developed CREAT (CRISPR‐based genome trimming with a multi‐homology‐arm template), a CRISPR‐based genetic approach that enables systematically classifying essential versus non‐essential genomic regions and subsequently deleting the latter category. Iterative application of CREAT facilitates the construction of microbial chassis cells with minimal genomes.

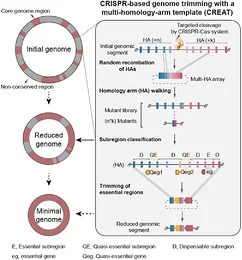

## 1 Introduction

Studies in the first wave of microbial genome sequencing in the 1990s revealed that only a small subset of genes is conserved across microorganisms. This immediately raised a fundamental question of what constitutes the minimal gene set required for sustaining life under defined conditions [1]. Subsequent efforts to construct a minimal genome have followed two main experimental paradigms: (a) “bottom‐up” synthesis of a minimal genome and (b) “top‐down” genome reduction [2, 3]. The former involves de novo assembly of different functional gene modules to produce a viable cell, as exemplified by the synthesis of the *Mycoplasma mycoides* JCVI‐syn3.0 genome [4]. The latter relies on iterative, “trial and error” deletion of non‐essential genomic segments, yielding genome‐reduced model strains including *Schlegelella brevitalea*, *Bacillus subtilis*, *Escherichia coli*, *Pseudomonas putida*, *Pseudomonas aeruginosa*, and *Corynebacterium glutamicum* [5, 6, 7, 8, 9, 10, 11, 12, 13, 14, 15].

Despite three decades of research, the minimal gene set required for life remains experimentally unresolved. Progress in both strategies has been slow, largely due to their reliance on empirical approaches. In the “top‐down” reduction, for instance, deletions are typically guided by essential genes identified through comparative genomic analyses, genome‐wide knockout screens, or transposon mutagenesis [16, 17, 18]. However, such studies often underestimate the number of essential genes [19, 20, 21, 22], as new essential genes can emerge under specific growth conditions—as observed in *Mycobacterium tuberculosis* [23], or through synthetic lethality resulting from the deletion of functionally redundant genes [24, 25]. Consequently, attempts frequently failed to delete genomic regions between previously‐defined essential genes [5, 15, 26, 27], and viable mutants were eventually obtained by progressively testing deletions of smaller fragments in a trial‐and‐error fashion. This process is not only laborious but also often incomplete, leaving residual non‐essential genomic fragments scattered among essential genes within the regions targeted for genome trimming analysis.

CRISPR‐Cas‐based genome editing has served as a versatile and precise platform for microbial genomic manipulation. CRISPR‐Cas systems are broadly classified into two classes and seven major types (Type I‐VII) based on their effector complexes and functional mechanisms [28]. Among them, type I CRISPR systems represent the most widespread type in bacteria and archaea, employing a multi‐subunit Cascade complex for target DNA recognition and the Cas3 helicase‐nuclease for processive DNA degradation [29, 30]. Notably, previous studies have demonstrated that Type I CRISPR‐Cas systems are capable of mediating large‐scale genomic deletions [5, 31, 32], a feature that renders them particularly suitable for top‐down genome trimming strategies.

To address the longstanding challenges in the top‐down genome reduction, we integrated CRISPR‐Cas facilitated genome targeting and “homology arm (HA) walking” to systematically distinguish essential from nonessential subregions within a given genomic segment. Non‐essential subregions are then precisely deleted and replaced with a synthetic cassette comprising the identified essential genes, yielding a trimmed genome in a single step. This approach, named CRISPR‐based genome trimming with a multi‐homology‐arm template (CREAT), has been successfully applied to the crenarchaeal model *Sa. islandicus* [33, 34, 35] and extended to two bacterial models: *B. subtilis* and *E. coli*.

## 2 Results

### 2.1 Classical CRISPR‐Based Large Genomic Segment Deletion Analysis Reveals Additional Essential Genes in the *Sa. islandicus* Genome

In a previous work, genome‐wide Tn5 insertion mutagenesis of *Sa. islandicus* M16.4 revealed 441 essential genes [18]. Most of these essential genes are clustered around the three replication origins [36] conserved among *Sulfolobales* organisms, and only a few are dispersed in the origin‐distal regions, which generates essential vs. non‐essential genomic segments in this archaeal genome (Figure S1A).

A. Locations of predicted essential genes across the *Sa. islandicus* REY15A genome, which is indicated by blue lines. The 6 largest predicted dispensable genomic segments are indicated as gS1–gS6. Sizes of the genomic segments are shown in parentheses. B. Schematic for the targeted large fragment deletion facilitated by the endogenous type IA CRISPR‐Cas system in the archaeon. The CRISPR RNA (crRNA) transcribed from the plasmid carrying the artificial mini‐CRISPR locus directs the Cascade protein complex to achieve targeted cleavage at the target site, and repair templates on the plasmid mediate template‐directed repair of the generated DNA double‐strand break, finally yielding mutant chromosomes with the target region deleted (Step 1). Genome‐edited transformants were selected on media lacking uracil (Uracil‐) (Step 2), and viable mutants were isolated on uracil‐supplemented media (Uracil+) to facilitate the loss of the pGE plasmid (Step 3). Flanking primer pairs gSn‐FP‐F and gSn‐FP‐R were used to detect the presence of mutated loci; Internal primers (gSn‐IP‐F/ gSn‐IP‐R) were used to detect an internal marker only present in the wild‐type strain. C. Bar graphs showing colony formation units after transforming the archaeal cells with different plasmids. The orange bars represent the transformation efficiency of genome‐targeting plasmids carrying a mini‐CRISPR array but no repair template (‐RT); the blue bars represent the transformation efficiency of genome‐editing plasmids (pGE) carrying both a mini‐CRISPR array and a repair template (+RT); the pink bars represent the transformation efficiency of control plasmids lacking both the mini‐CRISPR array and repair template. D. Bar graphs showing fractions of genome‐edited colonies and those of viable mutants. Data from 3 independent experiments are shown. Source data are also provided with this paper.

**FIGURE 1:**
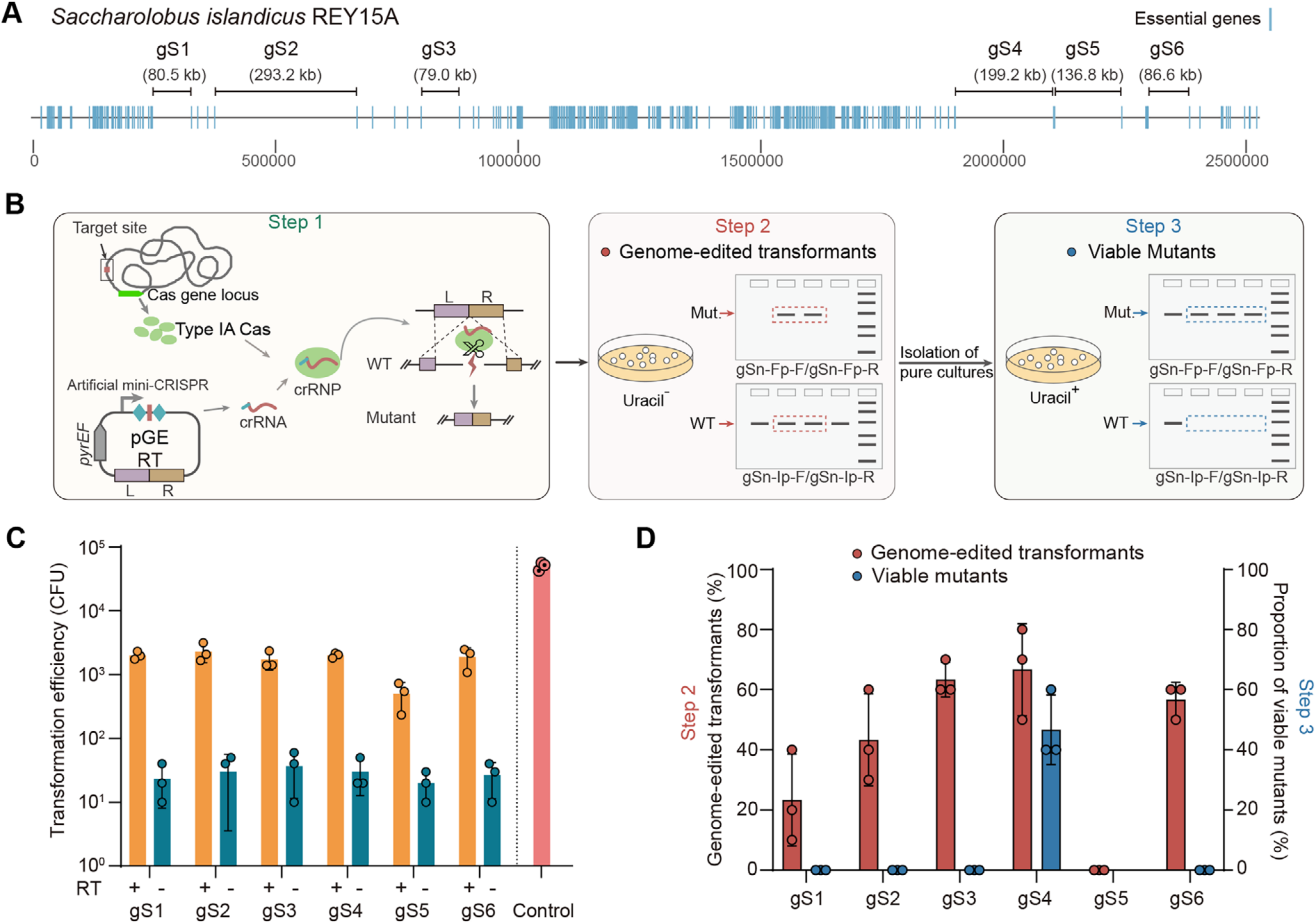
Classical target deletion analysis of predicted dispensable genomic regions using endogenous CRISPR‐based genome editing.

Comparative genomic analysis between *Sa. islandicus* M16.4 and *Sa. islandicus* REY15A revealed a high degree of genome synteny, indicative of the highly conserved gene order and genomic arrangement between the two genomes (Figure S1B). Six large non‐essential genomic regions appeared in the REY15A strain, ranging from 79 to 293 kb, which are referred to as gS1 to gS6 (Figure 1A and Figure S1C), and subjected to deletion analysis using the CRISPR‐based gene deletion method with the endogenous type IA CRISPR‐Cas of the archaeon as described previously [37, 38] (Figure 1B). Genome‐editing plasmids were constructed and electroporated into the cells of *Sa. islandicus* E233S (Step 1), a genetic host derived from *Sa. islandicus* REY15A with deletions of the *pyrEF* and *lacS* genes (Data S1), in which *pyrEF* encodes an orotate phosphoribosyltransferase and an orotidine 5’‐phosphate decarboxylase, whereas *lacS* encodes a β‐galactosidase. The *pyrEF* genes serve as a genetic marker to confer selection in the absence of uracil, while *lacS* is an indicator marker [39]. We found that transformation efficiencies with pGE plasmids carrying the repair template (RT) are 10‐ to 100‐fold higher than their corresponding genome‐targeting plasmids, mini‐CRISPR array‐carrying plasmids that facilitate autoimmunity (Figure 1C). Furthermore, PCR genotyping of their transformants indicated that the designed mutated DNA segments were present in the transformants of all pGE plasmids except for pGE‐gS5 (Step 2) (Figure 1D and Figure S2A,B), but the wild‐type (WT) chromosome was also present in all analyzed transformants (Figure S2A,B). At least 30 colonies of transformants were chosen for each genome‐editing plasmid and used in colony purification via multiple rounds of stripping for single colonies, and this revealed that mutated alleles eventually disappeared in the transformants of 4 plasmids, i.e., pGE‐gS1, ‐gS2, ‐gS3, and ‐gS6 (Step 3). Consequently, deletion mutants were only obtained with pGE‐gS4 (ΔgS4), carrying a 199 kb deletion in the mutated genome (Figure 1D and Figure S2F). These results clearly indicated that additional essential or quasi‐essential genes/synthetic lethal genes are present in the remaining 5 of the 6 tested genomic segments.

To test that, gS2, the largest region spanning 293 kb in the genome, was divided into three subregions named gS2‐1, gS2‐2, and gS2‐3 for conducting targeted deletion using the same CRISPR‐based genome‐editing method (Figure S2C). While mutants lacking the gS2‐2 region were readily obtained, colonies of transformants carrying the deletion of either gS2‐1 or gS2‐3 were not obtainable (Figure S2D,E). These results reinforced that the successful deletion of large genomic segments operates on a trial‐and‐error basis. However, these results also suggested that systematically redefining borders of deletable subregions in a given genomic segment would greatly facilitate the genome trimming process in any microbe.

### 2.2 Homology Arm Walking Enables Rapid Classification of Non‐Essential, Essential, and Quasi‐Essential Subregions in a Given Genomic Segment

**FIGURE 2:**
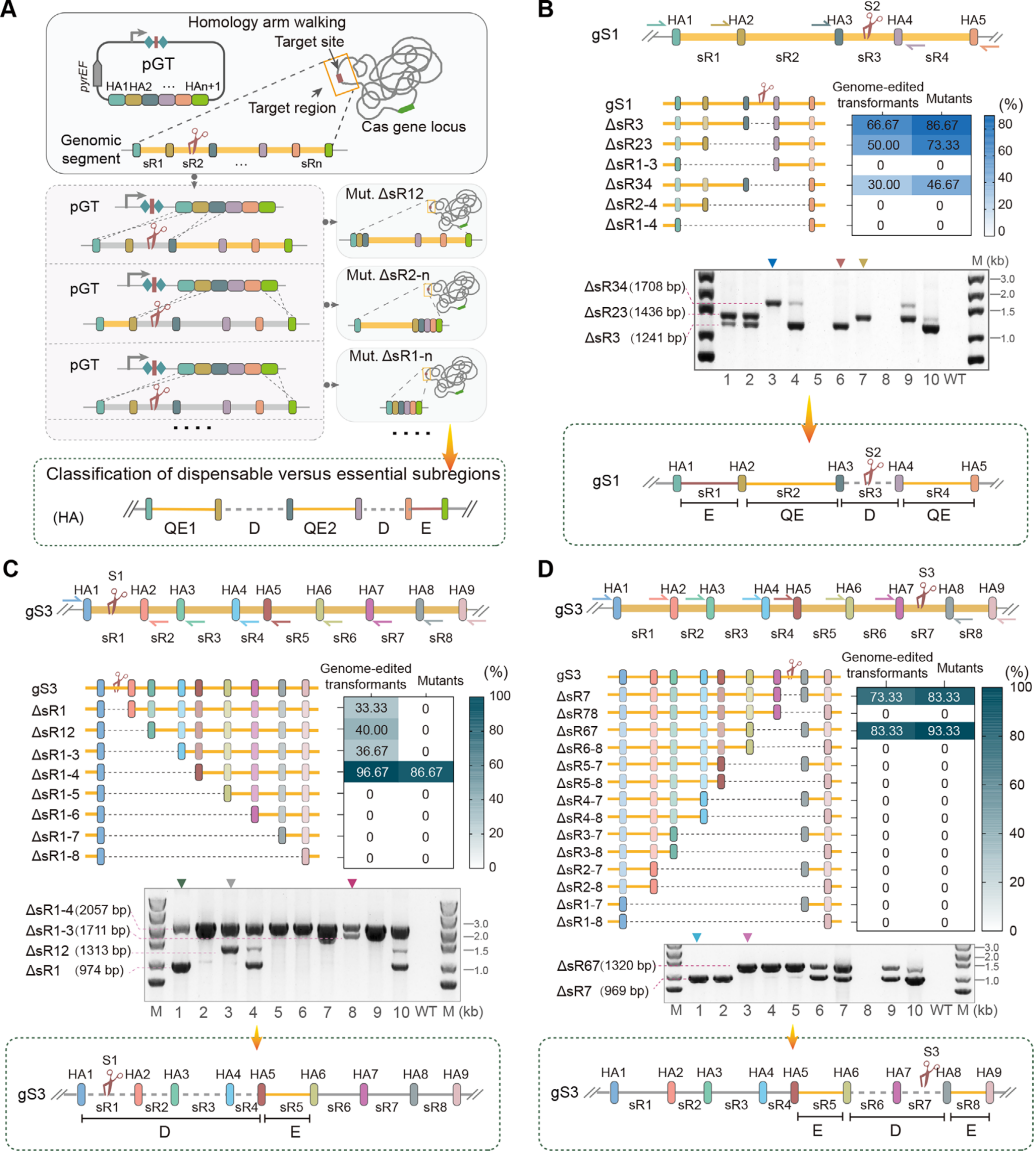
Classification of essential vs. non‐essential subregions in unknown genomic segments of *Sa. islandicus* using homology arm walking.

We then designed a homology arm (HA) walking method to systematically identify nonessential subregions in a given genomic segment. The HA walking design included the following steps: (a) dividing the genomic segment into several subregions (denoted sR1, sRn) subjected to deletion analysis by designing an intervening short DNA sequence (0.3–1 kb) as homology arms (denoted HA1 to HAn+1). Three criteria were applied to define subregion boundaries: (i) intact genes and operon structures were preserved without disruption; (ii) each subregion was set to 5–20 kb to balance HA walking efficiency and the accuracy of essentiality mapping; (iii) known regulatory elements, including promoters and terminators, were retained within a single subregion to avoid false results caused by interrupted regulatory signals. (b) all the HAs are assembled to yield a synthetic DNA fragment as repair template (RT), and (c) one or more targets were selected in appropriate subregions (e.g., sR2) for conducting the CRISPR‐based genome targeting in which double crossover recombination can occur between the chromosome targets and two HAs in any possible combinations on RT, and all HAs except for the leftmost and rightmost ones can either function as a left homology arm or a right homology arm (Figure 2A). Putting these HA combinations together, they extend away from the targeting site bidirectionally, which can be described as the bidirectional HA walking. Thus, HA walking from each targeting site allows the identification of two essential subregions, which can be identified by multiplex PCR scanning and sequencing.

A. Schematic of the CRISPR‐based homology arm (HA) walking with pGT plasmids in *Sa. islandicus*. Upon CRISPR targeting, HAs in the repair template (RT) are recombined with the corresponding fragments in the host genome in different combinations, yielding a repertoire of all possible recombinants carrying different deleted regions. Based on PCR identification and sequencing typing of the recombinants, each subregion in the target region can be classified as a dispensable subregion (D), or an essential subregion (E), or a quasi‑essential subregion (QE). N+1 denotes the total number of HAs; the number of the corresponding subregions is N (sR1 to sRn). B. HA walking analysis of the gS1 segment. Top: Diagram of gS1 with 5 HAs; Bottom: Gel image of PCR genotyping of transformants with pooled primer sets. Transformant colonies used for mutant purification are indicated by triangle symbols; Middle: Diagram showing genotypes of all theoretical recombinants with subregion deletion annotations (Left) and Results: The heatmap shows the frequency of mutant strains detected/obtained; Darker color indicates a higher frequency of occurrence. Values are means of three biological replicates. The dashed box shows a schematic diagram of the classification results for each subregion in the target region, as determined by mutant typing. The abbreviations D, E, and QE are defined the same as in Figure 2A. HA walking analysis of the gS3 segment was conducted with 3 different targeting sites, and their data are shown for targeting sR1 (C); sR7 (D), and sR4 (Figure S3F). Figure annotations are the same as in (B).

The gS1 region was the first segment to be analyzed by HA walking. It is 80.5 kb in size, containing 74 genes. gS1 was divided into 4 subregions (gS1‐sR1 to ‐sR4) of <20 kb in size, for which 5 HAs of 0.4 kb were designed. The designed HAs were fused together by SOE‐PCR to generate the repair template. Two target sites were selected: one on the left, i.e., in the gS1‐sR1 subregion, while the other, on the right, in gS1‐sR3 (Figure 2B). Since HA walking would stop before the subregion containing an essential gene from either direction, this assay would lead to the identification of two essential genes/gene blocks. Genome‐editing plasmid pGT‐gS1‐S1 and pGT‐gS1‐S2 (Data S1) were constructed, and plasmid transformation showed that only pGT‐gS1‐S2 yielded transformants (Figure 2B and Figure S3A,B). These results indicated that gS‐sR1 carries (a) essential gene(s). Transformants obtained with pGT‐gS1‐S2 were analyzed by multiplex PCR scanning followed by Sanger sequencing of PCR products. Notably, multiplex PCR with a pool of multiple forward and/or reverse primers revealed cells carrying deletions of different combinations of HAs in the same or different transformants, such as HA2‐HA4 (gS1‐ΔsR23), HA3‐HA5 (gS1‐ΔsR34), and HA3‐HA4 (gS1‐ΔsR3). The editing efficiencies of these recombinants ranged from 30% to 66.67%, and this variation is likely attributable to sequence‐specific biases associated with distinct homology arms. However, the deletion from HA2 to HA5 (gS1‐ΔsR2‐4) was not obtained (Figure 2B and Figures S3C and S4A). Thus, all mutants either retained gS1‐sR2 or gS1‐sR4, suggesting that these two subregions may carry redundant genes of essential function.

To verify the above observations, classical genome‐editing plasmids were constructed to target each nonessential subregion for deletion (Figure S3D). We found that deletions of gS1‐ΔsR23, gS1‐ΔsR34, and gS1‐ΔsR3 were obtained, but gS1‐ΔsR2‐4 was not, consistent with the data obtained from the HA walking, indicating that this method can be used to classify essential and nonessential genomic subregions. Strikingly, the HA walking analysis can also identify subregions carrying functionally redundant genes.

We then rigorously tested the HA walking analysis with gS3, a 79‐kb segment in the *Sa. islandicus* genome. This genomic region was evenly divided into 8 subregions with which 3 targeting sites were selected on gS3‐sR1 (left), gS3‐sR4 (middle), and gS3‐sR7, leading to the construction of pGT‐gS3‐S1, ‐S2, and ‐S3 (Data S1). HA walking was conducted with pGT‐gS3‐S1 transformation, and screening of colonies of transformants recovered diverse genome‐editing patterns, but the largest deletion removed the first 4 subregions (Figure 2C and Figures S3E and S4B), indicating gS3‐sR5 carries (a) essential gene(s). HA walking with pGT‐gS3‐S2 showed that the deletable region is still restricted to the first 4 subregions (Figure S3F), reinforcing the conclusion obtained with pGT‐gS3‐S1 walking. However, HA walking from the right end by using pGT‐gS3‐S3 targeting gS3‐sR7 recovered deletions of gS3‐sR6 and gS3‐sR7 (Figure 2D and Figure S4C). Together, HA walking revealed that gS3‐sR5 and gS3‐sR8 carry essential genes in the gS3 genomic region.

In addition, the transformants obtained with pGT‐gS3‐S1 were screened. We found that, as observed above, there were multiple editing patterns (Figure 2C). However, during the purification process, deletions spanning the first four sub‐regions (gS3‐sR1‐4) were dominant (with an efficiency of 86.67%), suggesting that the deletion of this genomic fragment might confer a competitive growth advantage. To verify the results, mutants were obtained using a genome editing method specific to the given genomic fragment, including gS3‐ΔsR1, gS3‐ΔsR12, and gS3‐ΔsR1‐3. These mutants were cultured with the parental strain gS3‐ΔsR1‐4. We found that the triple sub‐region deletion mutant grew faster than all other mutants (Figure S3G), exemplifying the application of the HA walking strategy in identifying superior mutant phenotypes.

### 2.3 Reconstruction of the Archaeal Genome by Replacing the Original Genomic Segment With a Synthetic Assembly of Essential Genes

**FIGURE 3:**
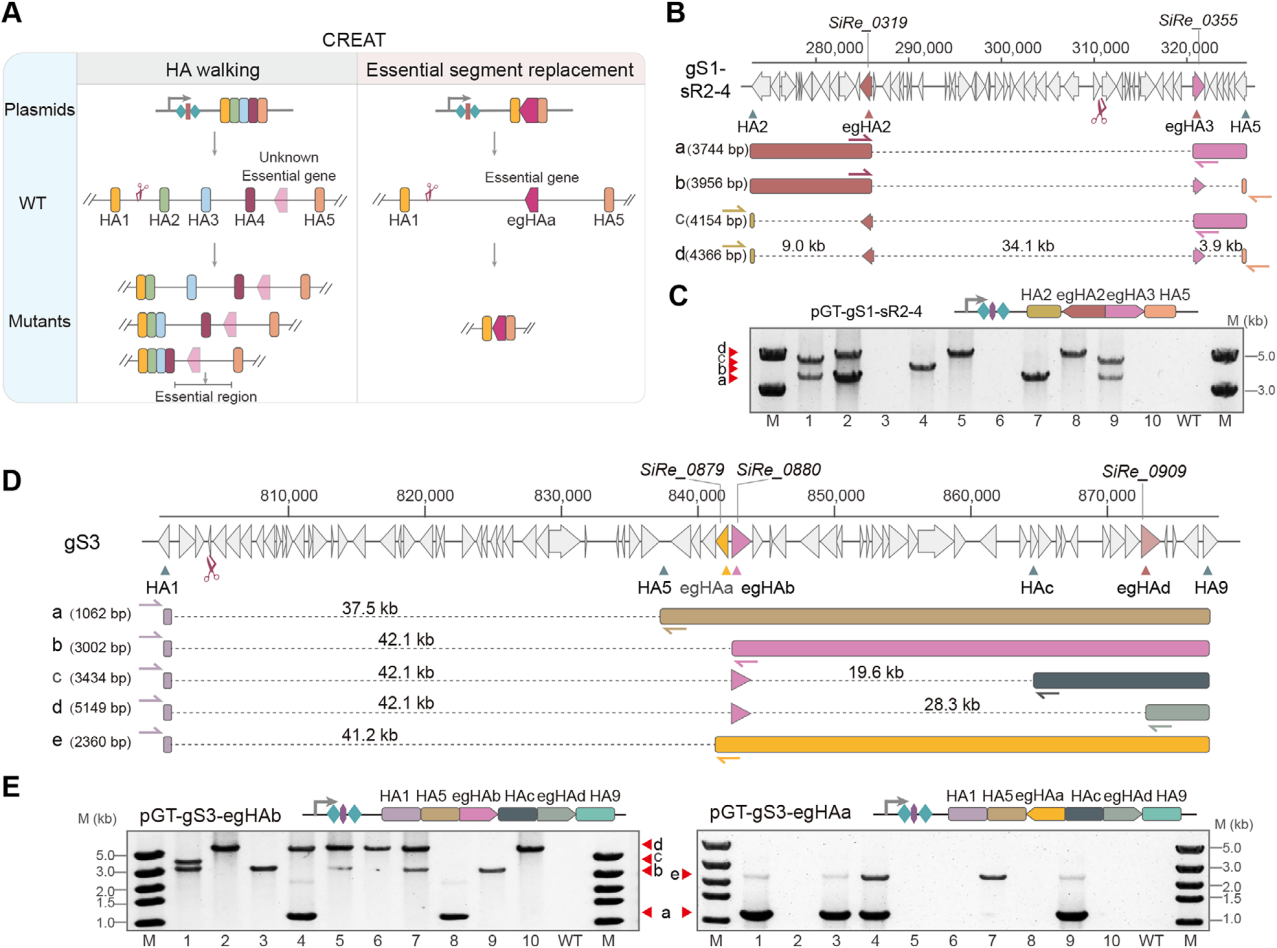
Replacing target genomic segments with a synthetic fragment of essential gene homology arms (egHAs).

The classification of essential and nonessential genomic subregions by the HA walking fostered the idea of conducting genome reduction by reconstituting the targeted genomic region with an assembly of the essential genes, enabling deletion of discontinuous non‐essential regions in one step. (Figure 3A). In order to do that, genes in the essential subregions were analyzed for their possible functions based on their annotations, we found that a few candidate essential genes, including: (a) *SiRe_0319* and *SiRe_0355*, which encode FAD‐type oxidoreductases that may participate in the electron transfer process and are of vital importance for maintaining normal cellular metabolism [40, 41] (Tables S1 and S2); and (b) two genes in gS3‐sR5, namely *SiRe_0879* (phosphoadenosine phosphosulfate reductase) and *SiRe_0880* (polysaccharide biosynthesis protein) (Table S3) could have an essential function since the former participates in sulfur metabolism, while the latter belongs to the MATE family of flippase (named archaeal glycosylation R, aglR), involved in lipopolysaccharide biosynthesis and the N‐glycosylation of S‐layer proteins [42]. These candidate genes were assembled together by PCR to yield essential gene homology arms (egHAs), including egHA2 (*SiRe_0319*), egHA3 (*SiRe_0355*), egHAa (*SiRe_0879*), and egHAb (*SiRe_0880*) (Figure 3B,D). Characterization of the transformants via multiplex PCR and Sanger sequence revealed the following results: (a) introducing either egHA2 or egHA3 or both essential genes into the tandem multi‐homologous arm array resulted in the removal of the gS1‐sR2‐4 region (Figure 3B,C and Figures S5 and S6A), and (b) including egHAb led to the successful replacement of the entire gS3 region with the designed egHAs, but replacement only with egHAa failed (Figure 3D,E and Figure S6B). Thus, essential gene complementation showed that while *SiRe_0319* and *SiRe_0355* are functionally redundant genes, conferring synthetic gene essentiality in gS1, *SiRe_0880* represents an essential gene identified for *Sa. islandicus* REY15A.

A. Schematic of the CREAT strategy. Nonessential genomic subregions were identified by HA walking analysis and refined by the prediction of new essential genes. Deletion of non‐contiguous nonessential genomic subregions is conducted by replacement of the target genomic segment with an assembly of essential genes. B. Gene essentiality analysis of the gS1‐sR2‐4 subregions. The locations of the two putative essential genes were highlighted in red and pink. Four possible outcomes are illustrated in a‐d with the deleted region shown in dot line, and their corresponding genotypes were identified in different transformants by PCR with pooled HA‐flanking primers, which are marked with the same letters and indicated with red arrows in C. RT with an assembly of HA2, egHA2, egHA3, and HA5 is shown above the gel image. D. Gene essentiality analysis of the gS3 region with the pGT‐gS3‐eHAa and pGT‐gS3‐eHAb plasmid. Six possible outcomes are illustrated as a‐e with the deleted region shown in dot line, and their corresponding genotypes were identified in different transformants by PCR with pooled HA‐flanking primers, which are marked with the same letters and indicated with red arrows in E. Each RT with an assembly of different HAs and egHAs is shown above the corresponding gel image.

To this end, we have established CRISPR‐based genome trimming with a multi‐homology‐arms template (CREAT), a strategy designed for an in‐depth analysis of essential genes in a genomic segment and trimming of nonessential subregions therein. The CREAT strategy includes classification of essential, quasi‐essential, and nonessential subregions by HA walking, prediction of new essential or quasi‐essential genes therein, and eventually testing the essentiality of putative essential genes and replacing the genomic segment for genome trimming with a reconstituted DNA segment of new essential and quasi‐essential genes (Figure 3A). The outcome is new strains with artificial genomic segments of only essential genes. By now, the application of this strategy in the genome trimming of 8 genomic segments in *Sa. islandicus* REY15A yielded a genome reduction of 20.8% (525.2 kb) from the wild‐type genome (Figure S7A and Table S4). Analysis of growth phenotypes in genome‐reduced strains demonstrated that deletions in specific genomic regions (e.g., region 481,619–564,744) result in significant growth impairment. Although the CREAT method enables the selection of strains exhibiting growth advantages, the Δ9 mutant with the most extensive genome reduction still does not attain a growth rate comparable to that of the wild‐type strain (Figure S7B).

### 2.4 Cas9‐Based CREAT Approach Facilitates Genome Trimming of Discontinuous Dispensable DNA Segments in *B. subtilis*

**FIGURE 4:**
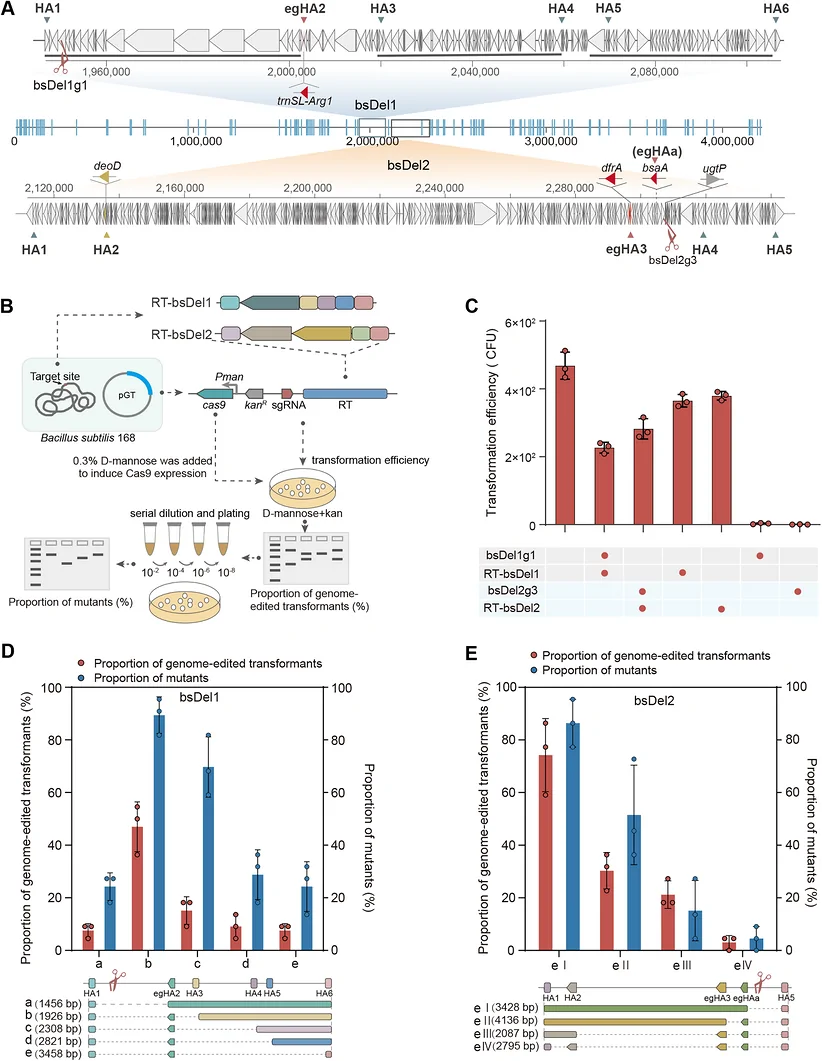
CREAT characterization of selected genomic segments of *B. subtilis*.

Examination of the genomic organization of *B. subtilis* 168 strain [43] and *E. coli* MG1655 strain [44] revealed similar modular structures (Figure S8A), prompting us to test whether the CREAT strategy developed for archaea can be applied to bacterial genome trimming. We aimed to develop a Cas9‐based CREAT method using *B. subtilis* as the host. Two genomic regions were chosen for CREAT genome trimming, including a well‐characterized 160 kb region and a less‐known 220 kb region in the *B. subtilis* genome (named bsDel1 and bsDel2, respectively) (Figure 4A). Three guide RNAs were designed for each region, i.e., bsDel1g1, ‐2, and ‐3 as well as bsDel2g1, ‐g2, and ‐g3, and they all were active in conferring the Cas9‐based self‐targeting (Figure S8B). Repair templates RT‐bsDel1 and RT‐bsDel2 were then generated as described in Materials and methods: the former contained six homology arms, one of which is the tRNA^Arg^ gene (egHA2), whereas the latter, 5 HAs with *dfrA*, an essential gene encoding a dihydrofolate reductase (Figure 4A). Insertion of the respective RT into the corresponding gRNA plasmids yielded a total of 3 genome‐targeting plasmids as listed in Data S1.

Two selected pGT plasmids, pGT‐bsDel1‐g1 and pGT‐bsDel2‐g3, along with their gRNA plasmids, were individually introduced into *B. subtilis* cells by natural transformation (Figure 4B) and scored for transformation efficiency. We found that the number of colonies produced with the former, a genome‐trimming plasmid, was 200‐fold higher than the latter, an interference plasmid (Figure 4C), indicative of successful genome editing with both pGT plasmids.

Transformants of pGT‐bsDel1‐g1 were then used for HA walking on bsDel1, and this revealed mutated alleles of all possible combinations (Figure 4D), consistent with the non‐essential feature of the protein‐encoding genes in this region. Furthermore, subsequent purification led to the isolation of mutants of all possible combinations, albeit with varying efficiencies. Notably, mutants carrying deletions between HA1‐HA3 (b) and HA1‐HA4 (c) were isolated at a higher frequency (>60%), relative to others (<30%), suggesting that deletion of these two regions may have less detrimental impact on the bacterial cell growth. These results indicated that CREAT also functions with the Cas9‐based genome editing.

HA walking on bsDel2 by targeting the middle subregion (HA3‐4) showed mutants carrying deletions between HA2 and HA5 (Figure S8C), suggesting the occurrence of (an) essential gene(s) in the first subregion (HA1‐2). However, HA walking on the same genomic segment by targeting the HA1‐2 subregion recovered mutants with deletion for HA1‐2 and HA1‐3 subregions, but lacking the subregion HA3‐4 contains (a) essential gene(s) (Figure S8D). Taken together, the above results indicate that HA1‐2 and HA3‐4 contain genes that produce synthetic lethality upon their deletion in *B. subtilis*. Examining annotations of the genes present in these two sub‐regions revealed two genes that function in oxidative stress defense, i.e., *sodC* and *bsaA*, encoding a superoxide dismutase and a bacillithiol peroxidase, respectively. To test if they could be functionally redundant, *bsaA* was included as a homology arm in the repair template for genome trimming, and this produced viable mutants carrying deletion of the bsDel2 region (Figure 4E, eIV). In addition, the bsDel2 region contains *ugtP*, a gene predicted as an essential one in the fatty acid synthesis pathway [45], which was shown to be dispensable for cell viability.

A. Location of two putative dispensable fragments, bsDel1 and bsDel2, as well as homology arms and target sites in the genome of *B. subtilis* 168. Known essential genes are highlighted in red triangles. B. Schematic of the genome‐editing process with a plasmid carrying tandem multi‐HAs and expressing Cas9‐gRNA. C. Colony formation units after transforming cells with different genome‐targeting and genome‐editing plasmids. The first group served as the empty vector control, lacking both guide RNA and repair template. Values represent means of three biological replicates. D. Bar graph showing the genome editing result with a pGT‐cas9 plasmid targeting the bsDel1 region. Letters a‐e denote mutant strains of different genotypes with a dotted line showing the deleted region. Values are means of three biological replicates. E. Genome editing result with a pGT‐bsDel2(*bsaA*)‐g3 plasmid targeting the bsDel2 region. eI‐IV denote genotypes of different mutant strains. Values are means of three biological replicates. Source data are provided with this paper.

### 2.5 Facilitating Cas9‐based CREAT Strategy by λ‐Red Recombineering in *E. coli*

Theoretically, the number of HAs in a repair template can be large; however, this number is limited by how long an RT can be, especially when the RT is to be cloned to a plasmid. In fact, the RT length limit constitutes a major constraint for the construction of the corresponding genome‐editing plasmids. Since λ‐Red recombineering facilitates highly efficient DNA recombination with short homologous DNA sequences, we attempted to incorporate the Red recombineering activity into CREAT (Figure S9A).

The CREAT‐Red system was first tested for HA walking on *E. coli* Ec17, a genomic region of ca. 70 kb only carrying nonessential genes. This region was divided into five subregions for which HAs of different sizes (50, 100, 200, 500 bp) were designed. These HAs were fused to yield repair templates of different HA sizes (RT50‐RT500) (Figure S9B,C). Notably, induction of λ‐Red expression increased transformation efficiency by more than 15‐fold, generating genome‐trimming mutants with greater diversity in HAs combinations relative to the uninduced condition. In addition, the length of HA also influenced the outcome of genome trimming since RT100 and RT200 generated 3–5‐fold more transformants than RT50 and RT500. Furthermore, RT200 generated all patterns of theoretical HAs combinations, whereas RT100 only produced a subset of them (Figure S9C,D). Together, these results indicated that RT200 is the optimal template to support the CREAT‐Red genome trimming in *E. coli*.

**FIGURE 5:**
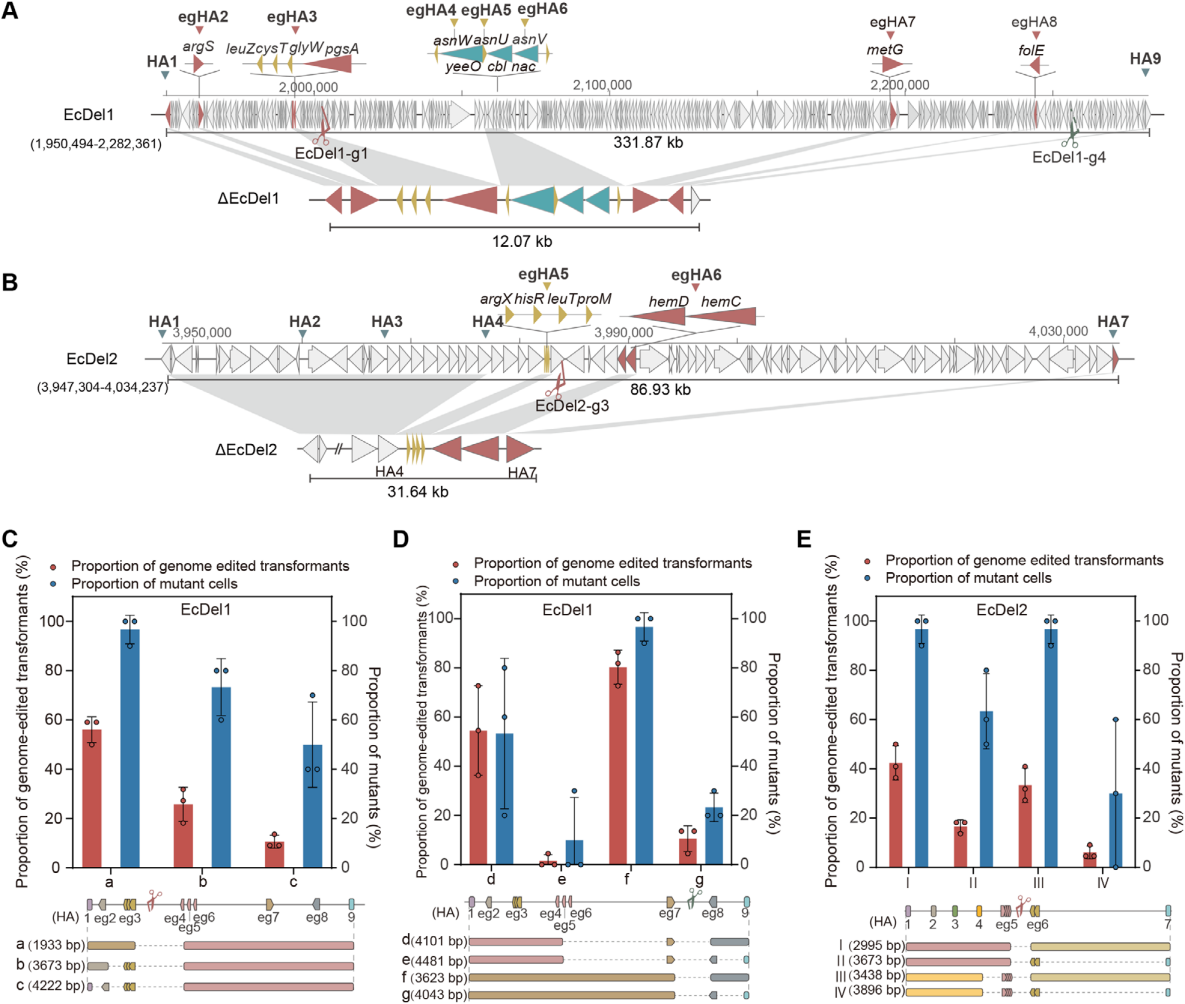
Enhancing the Cas9‐based CREAT strategy with λ‐Red recombineering in *E. coli*.

Two *E. coli* genomic segments, EcDel1 (331 kb) and EcDel2 (86.9 kb), carrying a few known essential genes (Figure 5A,B) were chosen for the application of the CREAT‐Red genome trimming in *E. coli* MG1655. Two linear repair templates were generated: the former contains 9 HAs, including 7 essential genes (egHA2‐8), while the latter contains 4 tRNA‐coding genes (fused as egHA5) and one essential gene (egHA6). Target sequences were selected within egHA3‐4 or egHA8‐HA9 of EcDel1 and egHA5‐6 of EcDel2, respectively, with which gRNA plasmids pEcDel1‐g1 or pEcDel1‐g4 and pEcDel2‐g3 were constructed (Data S1). The experiment was started by introducing each gRNA plasmid to *E. coli* cells along with the corresponding linear RT (Figure 5B). Transformants were obtained for transformations with both plasmids, which were analyzed by PCR scanning and sequencing to reveal their genotypes.

The first HA walking on EcDel1 was conducted by targeting the egHA3‐4 subregion, yielding mutants carrying a deletion for the first 3 subregions, i.e., between HA1 and egHA4, (Figure 5C). However, conducting a second HA walking on the same segment by targeting the egHA8HA9 subregion produced a deletion for egHA6 to HA9 (Figure 5D). The two deletable regions were then integrated together by using deletion mutants obtained from the first HA walking as host cells for conducting the second HA walking. The outcome of the second HA walking yielded the ΔEcDel1 synthetic DNA segment with an assembly of 13 essential genes (Figure 5A).

HA walking on EcDel2 by targeting the egHA5‐6 subregion yielded recombinant strains carrying HA4‐HA7 deletions, with the proportion of egHA5‐6 and egHA4‐6 region deletion mutants being over 90%, indicating a number of essential genes are present in the HA1‐HA4 subregion (Figure 5E). Indeed, HA1‐HA4 subregions contain 3 gene clusters, one with branched‐chain amino acid biosynthesis genes [46, 47]; another is the rho operon that regulates the transcription termination process in *E. coli* [48, 49]; the third includes genes involved in the polysaccharide synthesis of the ECA, an important component of the cell wall [50, 51]. In addition, we have also verified that the *wzxE*, *wecF*, *wzyE*, and *rffM* genes involved in the assembly and flipping process of the trisaccharide repeat unit of ECA synthesis (HA4‐eHA5 subregion) are dispensable in *E. coli* MG1655, as well as in *E. coli* CWG1573 [52]. In addition, CREAT analysis also integrated 2 known essential genes and four tRNA‐coding genes into the ΔEcDel2 fragments (Figure 5B). In summary, the application of the integrated CREAT‐Red strategy greatly facilitated rapid genome trimming across diverse microbial organisms.

A. Gene organization in EcDel1 segments in *E. coli* MG1655 and genome‐reduction strain. The location of the putative dispensable fragments, EcDel1, as well as the homology arms and target sites in *E. coli* MG1655 genome is described. Essential genes and tRNA are highlighted in red and yellow, respectively. Grey shadow shows the direct match of the EcDel1 segment between WT and ΔEcDel1. B. Genome structure map of the EcDel2 segment in *E. coli* MG1655 and genome‐reduction strain. C. Genome editing outcomes for deletion of the EcDel1 region with CREAT‐Red by GT‐EcDel1 and pEcDel1‐g1 plasmids. Values are means of three biological replicates. D. Genome editing outcomes for deletion of the EcDel1 region with CREAT‐Red by GT‐EcDel1 and pEcDel1‐g4 plasmids. Values are means of three biological replicates. E. Genome‐editing outcomes for deletion of the EcDel2 region with CREAT‐Red strategy. Values are means of three biological replicates.

**FIGURE 6:**
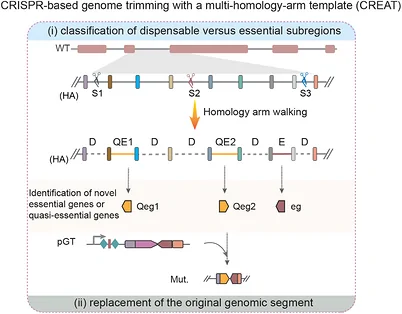
Schematic illustration of the genome trimming with CREAT strategy.

In conclusion, we have developed CREAT, a new genome‐trimming strategy with 2 successive steps, including (i) classification of dispensable versus essential subregions, (ii) identification of new essential genes therein, and replacement of the original genomic segment with a synthetic DNA fragment consisting of all identified essential genes (Figure 6). Together, this integrated genetic scheme provides the first method for systematically identifying all nonessential fractions and one‐step removing discontinuous dispensable regions in a given genomic segment, yielding a genome‐ reduced strain for iterative CREAT analyses of the required genomic segments, producing genome‐reduced strains with precisely designed deletions.

The two‐step integrated genetic scheme comprises: (i) systematic classification of dispensable and essential/quasi‐essential subregions through the HA walking method; (ii) targeted replacement of the original genomic segment with a synthetic DNA fragment construct harboring all identified essential genes or quasi‐essential genes within the validated essential or quasi‐essential subregions using genomic analysis. S1, S2, and S3 represent different targeting sites. HA: homology arm; E: essential subregion; QE: quasi‐essential subregion; D: dispensable subregion; eg, essential gene; Qeg: quasi‐essential gene.

## 3 Discussion

Herein, we report a streamlined genome‐trimming strategy that employs HA walking to systematically map gene essentiality and to reconstruct mutants carrying synthetic cassettes harboring newly identified essential genes. This approach enables precise classification of essential vs. nonessential regions within a targeted genomic segment, followed by targeted deletion of nonessential sequences and their replacement with assembled essential gene modules, yielding genome‐reduced strains in a single step.

Cells with a minimal genome have broad application potential spanning from fundamental research to industrial bioproduction. They serve not only as a unique window to explore the minimal requirements for life, but also as a high‐performance, streamlined, and functionally optimized chassis for biomanufacturing. However, the rational design of minimal genomes remains challenging, largely due to difficulties in accurately defining dispensable genomic regions. CRISPR‐Cas‐based targeted deletion has emerged as a powerful tool for genome reduction, which typically relies on a single pair of HAs located on both sides of the non‐essential region for templated repair to achieve precise deletion. However, the widespread presence of synthetic lethal gene pairs [4] and functionally ambiguous quasi‐essential genes [53] complicates the precise delineation of deletable regions, posing a major bottleneck in minimal genome construction. To address this challenge, conventional workflows often divide target regions into discrete subsegments and apply iterative, trial‐and‐error deletions—a process that requires extensive phenotypic and genotypic screening at each step (Figure S10A). In contrast, the CREAT strategy consolidates multi‐round functional assessment into a one‐step multiplexed procedure, directly yielding maximally trimmed or phenotypically optimized mutants (Figure S10B).

Recent advances in CRISPRi‐TnSeq [54] and dual Tn‐Seq [55] have enabled high‐throughput mapping of genetic interactions, informing targeted deletions of redundant or synthetic lethal genes. However, these genetic interaction networks require re‐evaluation after each reduction round due to context‐dependent shifts in gene essentiality. Although methods such as LoxTnSeq [56], which integrates TnSeq with the Cre‐loxP system for high‐throughput genome deletion screening, face limitations including non‐iterability, orientation‐dependent recombination outcomes, and procedural complexity. By comparison, the CREAT strategy supports iterative, multi‐round genome editing without prior gene essentiality screening at each step. And CREAT enables continuous and effective genome reduction by precisely identifying subregions that cause synthetic lethality within large target segments, even under dynamic genetic backgrounds.

Nevertheless, the CREAT approach also shows certain limitations. Since this strategy defines essential/non‐essential genomic subregions based on the failure to obtain viable recombinants, the lack of recombination between certain HAs due to their intrinsic bias in homologous recombination would raise uncertainty about whether the identified essential subregions are truly essential. In such cases, it is recommended to design new HAs for the questionable subregions to conduct parallel HA walking to remove the uncertainty about essential subregion classification. Furthermore, in the design of the CREAT strategy, we set the initial subregion size at 5–20 kb as a balanced starting resolution for the genome trimming with large‐scale subregions to prioritize reduction speed. However, uncertainty can also arise about whether the essentiality is determined by a single essential gene or multiple essential ones in an identified essential subregion. To address this issue, follow‐up CREAT experiments with high‐resolution HA pairs can be performed for a subregion of interest to precisely locate individual essential genes within the identified essential subregions, thereby eliminating residual non‐essential DNA in these regions and achieving deeper genome reduction. Finally, while CREAT can identify synthetic lethal gene pairs within the same targeted segment, synthetic lethal interactions between genes in different regions may lead to misclassification of the quasi‐essential genes as essential ones. In this case, functional redundancy of these genes can then be revealed by iterative CREAT analyses in different orders of targeted genomic regions in a microbial genome. Nevertheless, different deletion orders with CREAT still result in slight differences in the finally retained essential gene set, which may hinder the achievement of the theoretically absolute minimal genome.

We have partly addressed the above uncertainties by incorporating the phage‐derived λ‐Red recombination system to increase homologous recombination between HAs in *E. coli*. The finding that the CREAT‐Red scheme not only increased transformation efficiency but also generated more diverse HAs recombination patterns in *E. coli* (Figure S9C,D), encourages further experimentation with similar or different auxiliary recombination systems. In this regard, a recent study has demonstrated that the knockout of the *xseA* gene or the overexpression of the *xseB* gene boosts the recombination efficiency in multiple bacterial strains [57]. Furthermore, RecET‐like recombinases, YqaJ and YqaK have been implicated in enhancing recombination efficiency in *B. subtilis* [58]. These factors would further facilitate CREAT in recombination‑limited hosts, broadening its utility across diverse microbial systems.

## 4 Methods

### 4.1 Strains and Growth Conditions

*Sa. islandicus* E233S is derived from *Sa. islandicus* REY15A carrying the deletion of *pyrEF* and *lacS* genes [39]. *Sa. islandicus* strains were grown at 76°C in SCV media [39] or USCV media (uracil is supplemented to 20 µg/mL if required) with shaking at 150 rpm. *E. coli* MG1655 and *B. subtilis* 168 were cultured with Luria‐Bertani (LB) medium (Solarbio, Beijing, China) or LB agar plates (1.5% agar) supplemented with required antibiotics at 37°C or 30°C, respectively.

### 4.2 Comparative Genome Analysis

Genome collinearity analysis was performed using Mauve for the whole‐genome alignment between *Sa. islandicus* REY15A and *Sa. islandicus* M.16.4 genomes. Among them, the M.16.4 genome is used as the reference. Progressive alignment was carried out with default parameters (seed sequence length = 15, minimum LCB weight = 3). The algorithm automatically identified Locally Collinear Blocks (LCBs), and only those with a length of ≥ 10 kb and containing ≥ 5 conserved genes were retained for further analysis. Genome rearrangement events were inferred based on the positional offsets and directional changes of the blocks in the alignment results. Finally, the collinearity map was visualized using Mauve's built‐in visualization module [59]. The correspondence between the *Sa. islandicus* REY15A and M.16.4 genes were established using the MUMmer tool [60] and subsequently validated through the BLASTP tool.

### 4.3 Preparation of Tandem Multi‐Homologous Arm Arrays

Each homologous arm fragment (HA1, HA2, …, HAn) was amplified by PCR using Phanta Max Super Fidelity DNA Polymerase (Vazyme). The resulting PCR products were purified, and equimolar amounts of the purified DNA fragments were mixed with high‐fidelity DNA polymerase (without added primers) and subjected to 12–18 cycles of fusion PCR. This process utilized the fusion of adjacent homologous arms to generate fusion products containing varying numbers of homologous arms (HA1‐HA2, HA2‐HAn, …, HA1‐HAn). Finally, the fusion products were further amplified by PCR using specific primers at both ends of the multi‐HA array fragment, thereby enriching the desired target PCR product (HA1‐HAn). The short homologous arm arrays with lengths of 50 bp (RT50) and 100 bp (RT100) used in *E. coli* were obtained through direct DNA synthesis.

### 4.4 Plasmid Construction

#### 4.4.1 Archaeal pGE and pGT Plasmids

The procedure for constructing pGE plasmids targeting non‐essential regions was described previously [38]. A spacer was designed following a CCN PAM within the nonessential region, and the spacer fragment was generated by annealing of the corresponding complementary oligonucleotides. The spacer fragment was inserted into pSe‐Rp at the LguI restriction site, yielding pCRISPR plasmids carrying an artificial mini‐CRISPR array. Subsequently, the DNA fragment containing the repair template was cloned into the SphI‐XhoI sites of pCRISPR plasmid, yielding the gene‐editing plasmid pGE. Construction of pGT plasmid follows a similar procedure, except that the repair template (Tandem multi‐homologous arm array) was prepared by fusion of multiple individual homology arms.

#### 4.4.2 *B. subtilis* Interference Plasmids

pJOE8999 is a well‐established shuttle vector in which the expression of SpCas9 is induced by mannose and is widely utilized for gene editing studies in *B. subtilis* [61]. To construct the *B. subtilis* interference plasmid based on pJOE8999, a 20‐nt sequence upstream of the NGG motif within the target region was selected as the target sequence. Two complementary oligonucleotides with BsaI restriction enzyme‐compatible overhangs at their 5' ends were designed according to this sequence. These oligonucleotides were mixed in equimolar amounts, denatured at 95°C for 10 min, and then slowly cooled to room temperature for oligo annealing. Subsequently, the annealed DNA fragment was ligated into the BsaI‐linearized pJOE8999 vector using T4 DNA ligase at 22°C overnight, giving the pJOE8999‐gRNA interference plasmid.

#### 4.4.3 *B. subtilis* pGT Plasmids

The pJOE8999‐gRNA vector was linearized through double digestion with SalI and XbaI restriction enzymes. Subsequently, the purified tandem multi‐homology arm array was seamlessly integrated into the linearized vector using Gibson assembly technology. This resulted in the successful construction of the plasmid pGT‐bsDel.

#### 4.4.4 *E. coli* pP_tet_‐cas Plasmid

A dual‐plasmid Cas9‐based gene editing system developed for *E. coli* [62] was employed in this work, comprising pP_tet_Cas9 for hydrotetracycline‐inducible Cas9 protein expression and pEcgRNA plasmid (Addgene number: 166581) for generating gRNAs targeting different genomic loci. The pEcCas plasmid (Addgene number: 73227) contains multiple functional components. Among them, Pcas‐Cas9 expresses the SpCas9 protein with a constitutive promoter, while the ParaB‐gam/bet/exo module controls the expression of the λ‐Red recombination system via an arabinose‐inducible promoter. To engineer a plasmid enabling inducible Cas9 expression, the constitutive promoter within the Cas9 expression cassette of the pEcCas plasmid was substituted with a tetracycline‐inducible promoter. This modification allows for the precise control of Cas9 protein expression through the addition of anhydrotetracycline.

#### 4.4.5 *E. coli* Interference Plasmids

For the targeted knockout region, a 20‐nucleotide sequence immediately upstream of the NGG PAM motif was selected as the gRNA target recognition site. Based on this sequence, two complementary oligonucleotides were designed and synthesized, featuring 5’‐TAGT‐3’ and 5’‐AAAC‐3’ overhangs at their 5' ends to ensure specific complementarity with the BsaI restriction enzyme cleavage site. The oligonucleotides were mixed in equimolar amounts, subjected to thermal annealing at 95°C for 10 min, and then gradually cooled to room temperature to form a double‐stranded gRNA functional module. This module was subsequently ligated into the BsaI‐linearized pgRNA vector backbone using T4 DNA ligase, resulting in the pgRNA recombinant plasmid with specific interference capability.

### 4.5 Transformation Procedure

#### 4.5.1 *Sa. islandicus* Transformation

The preparation of *Sa. islandicus* competent cells and electroporation transformation procedures were implemented as previously described [39].

#### 4.5.2 *B. subtilis* Transformation

The preparation procedure for competent cells of the *B. subtilis* 168 strain is described as follows. A single colony was inoculated into 2.5 mL of growth media (GM) I (Spizizen minimal medium [63] with 1.0 g/L Yeast extract, 0.2 g/L Casein acid hydrolysate, 5.0 g/L Glucose) and incubated at 30°C with shaking at 150 rpm for 12 h. Subsequently, the culture was transferred to 10 mL of fresh GM I at a 10% inoculation volume and further incubated at 37°C with shaking at 200 rpm for 210 min. Following this, the culture was transferred again to 50 mL of GM II (GM I supplemented with 55.5 mg/L CaCl_2_ and 238.0 mg/L MgCl_2_) [64] at the same inoculation ratio and incubated under identical conditions for 90 min. Cells were harvested by centrifugation at 5000 rpm for 5 min, after which the supernatant was discarded, and the pellet was resuspended in 10 mL of GM II. The resuspended cells were then aliquoted into 100 µL portions per tube and either stored at −80°C or directly used for transformation experiments. The natural transformation of *B. subtilis* was performed as follows. Pre‐prepared competent cells of *B. subtilis* 168 were retrieved from −80°C storage and allowed to slowly thaw on ice. Subsequently, 1 µg of plasmid was added to the competent cells, followed by gentle mixing. The mixture was then incubated at 37°C for 30 min. Then, 0.3% D‐mannose and 200 µL of LB medium were added to the system, and the solution was further incubated at 37°C for 60 min. Finally, the bacterial suspension was evenly spread onto LB solid medium supplemented with kanamycin (5 µg/mL) and 0.3% D‐mannose, and then incubated at 30°C for 18–24 h.

#### 4.5.3 *E. coli* Transformation

The pP_tet_Cas9 was introduced into the MG1655 cells as previously described [62]. The preparation procedure for competent cells of the *E. coli* MG1655 with pP_tet_Cas9 is described as follows. Single colonies from kanamycin‐resistant LB agar plates were selected and inoculated into 5 mL of LB liquid medium supplemented with 50 µg/mL kanamycin. The culture was grown overnight at 37°C with shaking at 200 rpm. Subsequently, the culture was transferred to fresh LB medium containing 10 mM L‐arabinose (final concentration) and 50 µg/mL kanamycin at a 1% inoculation volume (v/v). Anhydrotetracycline was added as required to achieve a final concentration of 100 ng/mL. The culture was incubated at 37°C with shaking at 200 rpm for 2–3 h until the OD_600_ reached 0.4‐0.6. The culture flask was then immediately placed in an ice‐water bath for 10 min to terminate cell growth. Bacterial cells were harvested by centrifugation at 6000 rpm for 10 min at 4°C and washed twice with pre‐chilled sterile water under the same centrifugation conditions. The pellet was resuspended in 30 mL of pre‐chilled 10% (v/v) glycerol solution and re‐centrifuged at 6000 rpm for 15 min at 4°C. After discarding the supernatant, the bacterial pellet was resuspended in 3 mL of pre‐chilled 10% glycerol solution, aliquoted into 50 µL portions, and stored at −80°C for long‐term preservation.

Electroporation was employed for *E. coli* MG1655 transformation [62]. A mixture of 100 ng of the pEcgRNA plasmid, 0.5‐2.0 µg repair template (0.5 µg of RTs were used for Ec17 trimming, and 2.0 µg of GT‐EcDel1 or GT‐EcDel2 were used for EcDel1 or EcDel2 trimming, respectively), and competent cells was incubated on ice for 10 min. The mixture was subsequently transferred to a 2 mm electroporation cuvette and subjected to electroporation at 2.5 kV, 25 µF, and 200 Ω. Immediately following electroporation, 900 µL of antibiotic‐free LB liquid medium was added to the cells, and incubated at 37°C with shaking at 200 rpm for 4 h. After incubation, the bacterial suspension was evenly spread onto LB solid medium supplemented with kanamycin (50 µg/mL) and spectinomycin (100 µg/mL). Depending on the experimental design, additional inducers were included as needed: 100 ng/mL anhydrotetracycline (for Cas9 induction) or 10 mm L‐arabinose (for λ‐Red recombination system induction).

### 4.6 Mutant Screening and Purification

Electroporated *Sa. islandicus* cells were plated by double‐layer plating onto 0.7% Gelrite SCV plates, in which the transformants could be selected by uracil dropout selection [39]. And then, mutants were screened by PCR amplification with flanking primers generating the 1∼5 kb target products in size, while no PCR products were obtained in the wild‐type strains, in which the expected product exceeds the maximum amplification size under the PCR condition. The resulting PCR products were analyzed by agarose gel electrophoresis and by DNA sequencing (Tsingke, Qingdao, China). Purification of mutants is accomplished by serial dilution of the transformant colony suspension and plating. Mutants were screened by PCR genotyping with flanking (gSn‐Fp‐F/ gSn‐Fp‐R and internal (gSn‐Ip‐F/ gSn‐Ip‐R) primers.

Multiple tiling PCR, which is a PCR reaction in which more than two pairs of primers are added to the same PCR reaction system to simultaneously amplify multiple DNA fragments, was used to screen mutants produced by the CREAT assay. In our PCR reaction system, only one forward (F) or reverse primer (R) and multiple reverse or forward primers with the same total molar number are added (i.e., moles of F = moles of R1+R2…+Rn or moles of F1+F2…+Fn = moles of R). DNA visualization in the gel was conducted on a ZF‐288 instrument (Shanghai JP Analytical Instrument Co., Ltd, Shanghai, China) at 300 nm.

### 4.7 Statistics and Reproducibility

For genotyping, colonies were randomly picked to ensure they were representative of the overall colony population. The quantitative data presented in each figure were derived from three biologically independent experiments. Results were expressed as the mean ± standard deviation. In the data analysis, all data points were included, and no data were excluded. The essential gene information of all species was obtained from the update of the Database of Essential Genes (DEG 15) [65]. The genome alignment results were visualized using the BLAST Ring Image Generator tool [66]. Manual deletion of misrepresentations produced by the BRIG was carried out to reflect the actual genome results.

## Author Contributions

Q.S. conceived the study. G.Y., Z.G., Y.Q., Y.Z., X.T., X.F., and Q.S. designed the experiments. G.Y., Z.G., Y.Q., Y.Z., and P.Z. performed the experiments. G.Y., Z.G., Y.Q., Y.Z., X. T., X.F., and Q.S. conducted formal analyses of research data, G.Y. and X.F. wrote the first version of the manuscript. G.Y. prepared all the figures. Q.S. revised the manuscript with the help from G.Y. and X.F. All authors read and approved the final version of the manuscript.

## Funding

This work was supported by the National Key R & D Program of China (grant number 2023YFC3402003 to FX, grant number 2020YFA0906800 to QS), Department of Science and Technology of Shandong Province (project number ZR2024QC306 to GY), Department of Education of Shandong Province (project number 2023KJ009 to XF), Qingdao Natural Science Foundation (project number 25‐1‐1‐250‐zyyd‐jch) and SKLMT Frontiers and Challenges Project (SKLMTFCP‐2023‐05). Funding for open access charge: National Key R & D Program of China.

## Conflicts of Interest

Several patent applications have been filled by Shandong University relating to this work.

## Supporting information




**Supporting File 1**: advs76042‐sup‐0001‐SuppMat.docx.

**Supporting File 2**: advs76042‐sup‐0002‐DataSet.zip.

## Data Availability

The data that support the findings of this study can be found either in the article file, the Supplementary Information file, or the Supplementary Data files. Source data are provided with this paper.
