# Supplementary material for: CREAT: A CRISPR‐Based Genome Trimming Strategy for Systematic Identification of Dispensable Regions and Rapid Genome Reduction
Source: Adv Sci (Weinh). 2026 Jun 29:e76042. Online ahead of print. doi: 10.1002/advs.76042 (PMC13337030; doi:10.1002/advs.76042)
Supplement: Supplementary file 1 — Supporting File 1: advs76042‐sup‐0001‐SuppMat.docx. [file ADVS-9999-e76042-s002.docx]

**Supplementary information for**

**CREAT: A CRISPR-Based Genome Trimming Strategy for Systematic Identification of Dispensable Regions and Rapid Genome Reduction**

Guanhua Yuan^1^, Zhe Gao^1, 2^, Yingxuan Qi^1^, Yang Zhang^1^, Xuhui Tian^1^, Pengpeng Zhao^1^, Xu Feng^1^* and Qunxin She^1^*

1 CRISPR and Archaea Biology Research Center, State Key Laboratory of Microbial Technology, Shandong University, Qingdao, 266237, China

2 Current address: State Key Laboratory of Microbial Metabolism, School of Life Sciences and Biotechnology, Shanghai Jiao Tong University, 800 Dongchuan Road, Minhang District, Shanghai, 200240, China

* To whom correspondence should be addressed. Email: [shequnxin@sdu.edu.cn](mailto:shequnxin@sdu.edu.cn); fengxu@sdu.edu.cn.


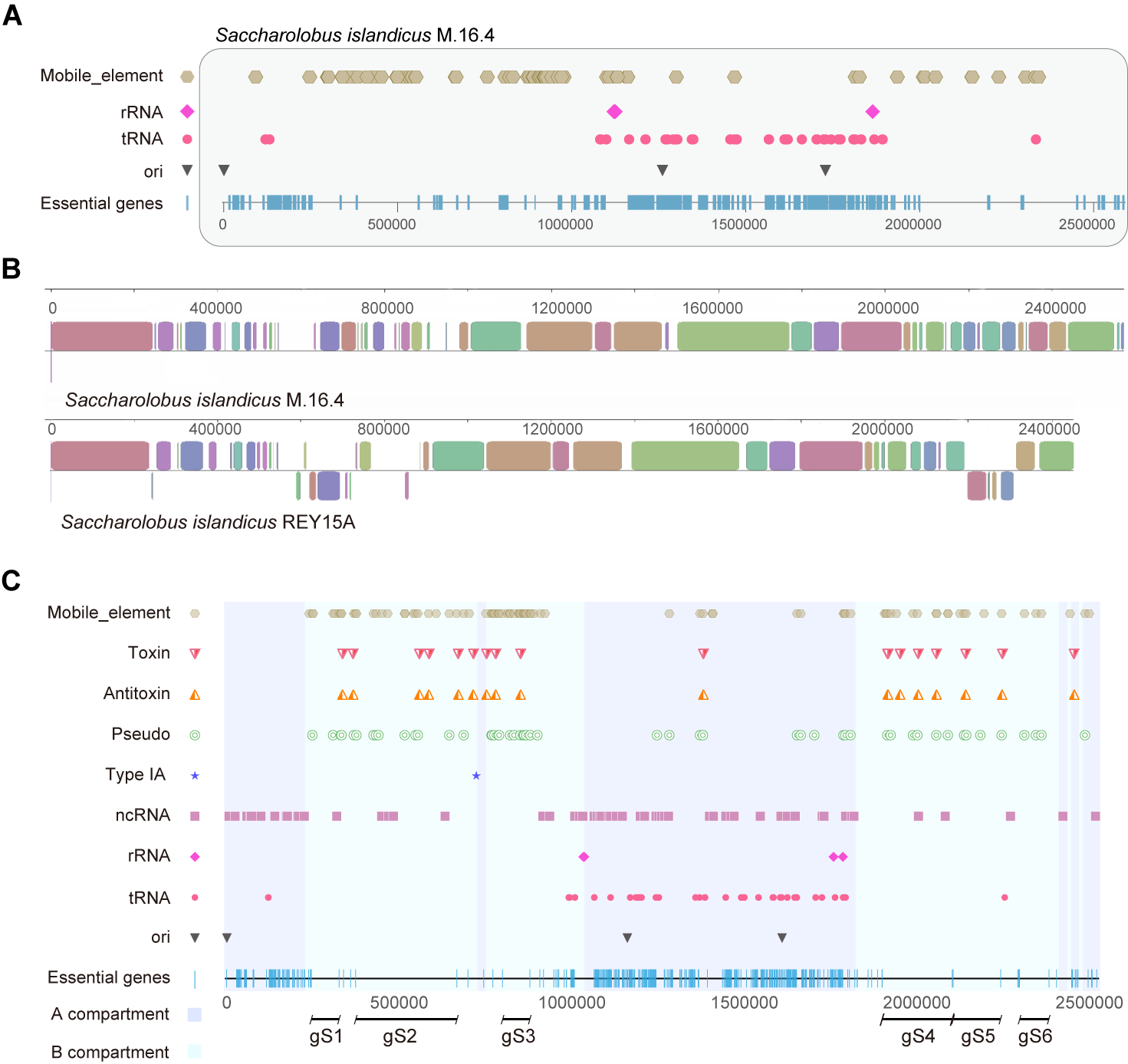


**Supplementary Figure S1. Genomic characteristics of *Saccharolobus islandicus***

**A.** Essential gene maps of *Sa. islandicus* M.16.4. Known essential genes, rRNA and tRNA genes, mobile genetic elements and replication origins (*ori*) of *Sa. islandicus* M.16.4 ^1^ are highlighted with the symbols shown on the left panel of the figure. **B.** Whole genome alignment of *Sa. islandicus* REY15A and *Sa. islandicus* M.16.4 by Mauve. Boxes of identical colors represent local colinear blocks (LCB), indicating homologous DNA regions shared by two genomes without sequence rearrangements. **C.** Genomic locations of predicted essential genes, A compartment (highlighted in grey), B compartment (highlighted in light cyan), toxin genes, anti-toxin genes, pseudogenes, mobile elements, ncRNAs, tRNAs, rRNAs, replication origins and genes coding for the type IA CRISPR-Cas system. The A or B compartment is the compartmentalized organization formed by the aggregation of chromosomes through "compartment domains", representing transcriptionally active or transcriptionally inactive regions, respectively. gS1-gS6 indicate the location of the six putative non-essential genomic segments.


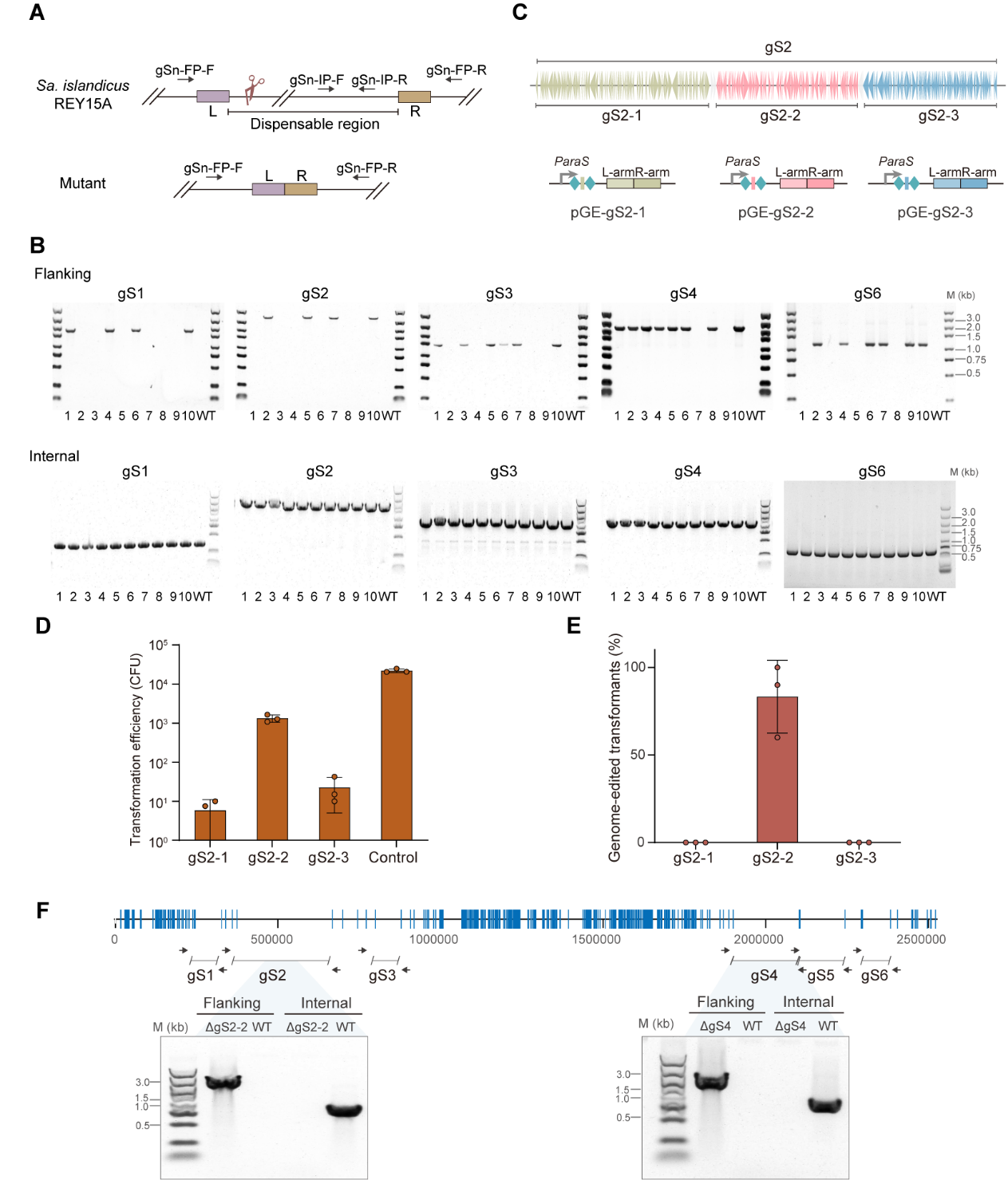


**Supplementary Figure S2.** **Classical “trial-and-error” deletion of putative dispensable regions with Type IA CRISPR-Cas3 in *Sa. islandicus***

**A.** Schematics of primer pairs used to verify the genotypes of genome editing mutants. Flanking primer pairs gSn-FP-F/gSn-FP-R are used to verify the genotype of mutants. Internal primer pairs gSn-IP-F/gSn-IP-R are used to verify the genotype of the wild-type strain. **B.** Genotyping results of different transformants with flanking primers and internal primers. 1-10 denotes 10 randomly picked colonies. **C.** Schematics of gene editing plasmids targeting different regions of the gS2 segment. Genome editing plasmids targeting three subregions gS2-1, gS2-2 and gS2-3, were constructed, yielding pGE-gS2-1, pGE-gS2-2 and pGE-gS2-3, respectively, which were used for exploring non-essential regions in the gS2 segment. **D.** Transformation efficiency of pGE-gS2-1, pGE-gS2-2 and pGE-gS2-3 plasmids. **E.** The Bar graph shows the proportion of genome-edited transformants. (f) Targeted deletion of gS2-2 and gS4 segments. Flanking and Internal denote the PCR amplicons generated with the flanking primers and internal primers, respectively.


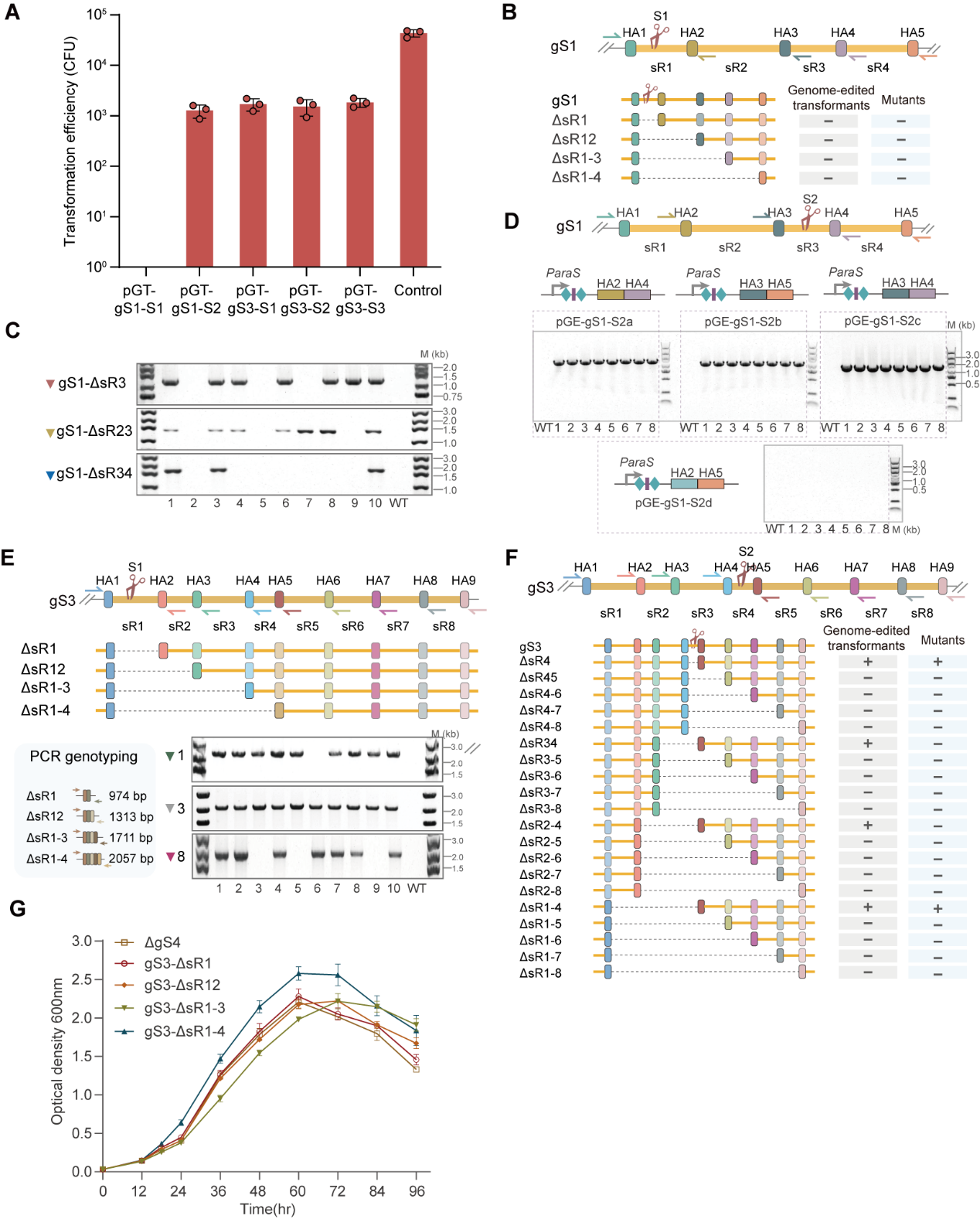


**Supplementary Figure S3.** **Defining nonessential genomic segments of *Sa. islandicus* with HA walking method**

**A.** Bar graphs showing colony formation units after transforming the archaeal cells with different pGT plasmids. **B.** HA walking analysis of the gS1 segment using the target site S1. The CRISPR target site is located within gS1-sR1, positioned between the first and second homology arms. Diagram showing genotypes of all theoretical recombinants with subregion deletion annotations (Left) and Results: “+” indicates mutant strains detected/obtained; “-” indicates mutant strains undetected/unavailable. **C.** The gel image presents the genotyping results of transformants carrying the pGT-gS1-S2 plasmid. The triangle symbol indicates the transformant colonies used for the further isolation process in Figure. 2b. **D.** Classical genome editing plasmids targeting different subregions of the gS1 segment. Genome editing plasmids targeting four subregions gS1-sR23, gS1-sR34, gS1-sR3 and gS1-sR2-4, were constructed, yielding pGE-gS2-S2a, pGE-gS2-S2b, pGE-gS2-S2c and pGE-gS2-S2d, respectively, which were used for verifying whether each subregion could be deleted. The homologous arms used by each classical genome editing plasmid are the same as those used in the HA walking experiment. **E.** The gel image presents the genotyping results of transformants carrying the pGT-gS3-S1 plasmid. The triangle symbol and numbers indicate the transformant colonies used for the further isolation process in Figure. 2c. PCR genotyping shows the PCR products and sizes amplified by the flanking primers of different mutants. **F.** HA walking analysis of the gS3 segment using the target site S2. The CRISPR target site is located within gS3-sR4. **G.** The growth profiles of ΔgS4 and different mutants. Data are presented as mean values ± SD. n=3 biologically independent samples.


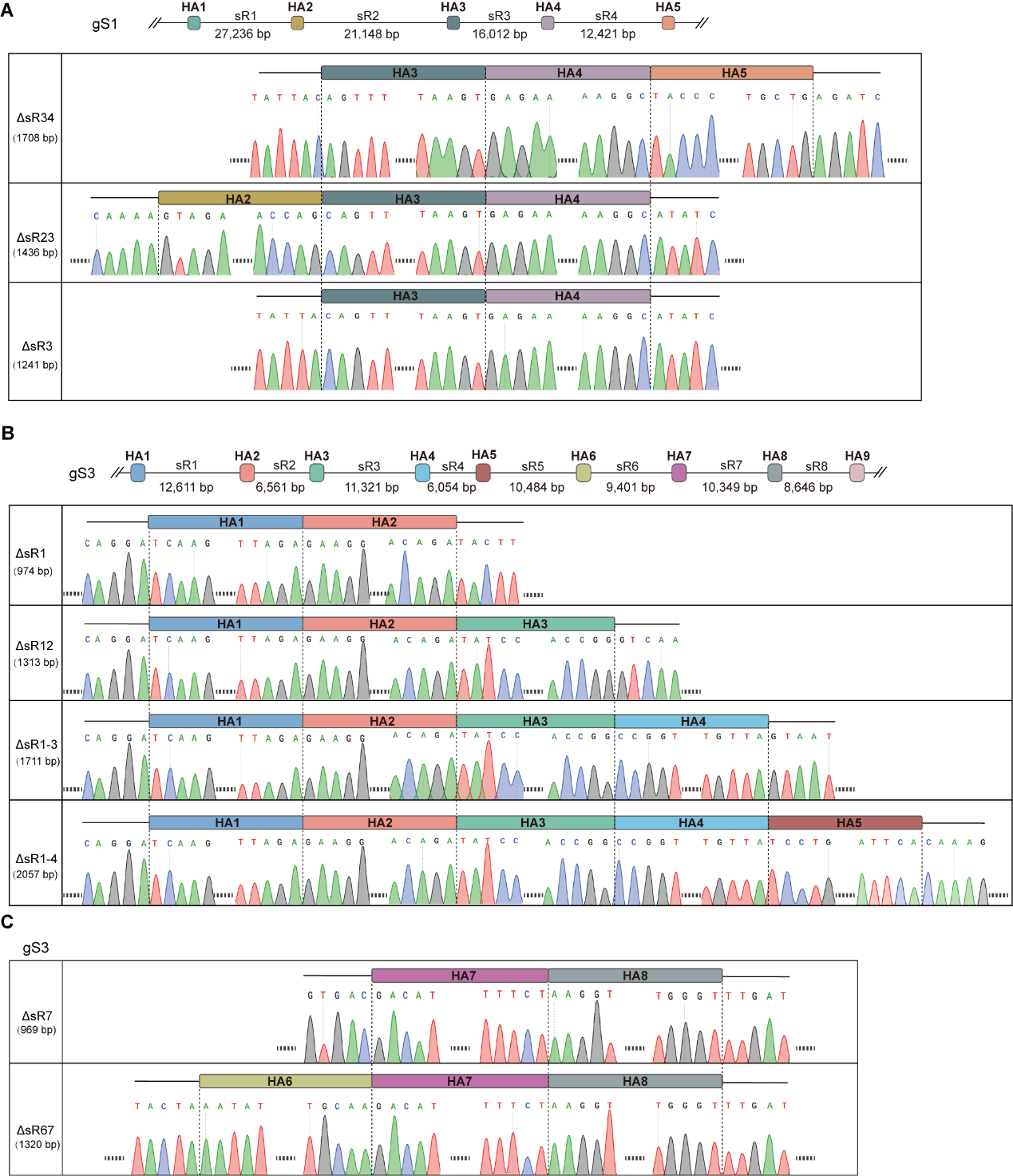


**Supplementary Figure S4. Validation of transformants generated by HA walking using Sanger sequencing**

Sanger sequencing results of each PCR product band after gel extraction from the PCR genotyping of gS1 **(A)** and gS3 **(B) (C)** transformants in Figure 2. The dashed black line indicates the unshown sequence results. The schematic diagram illustrates the positions of each homologous arm within the wild-type strain and the corresponding sizes of individual sub-regions.


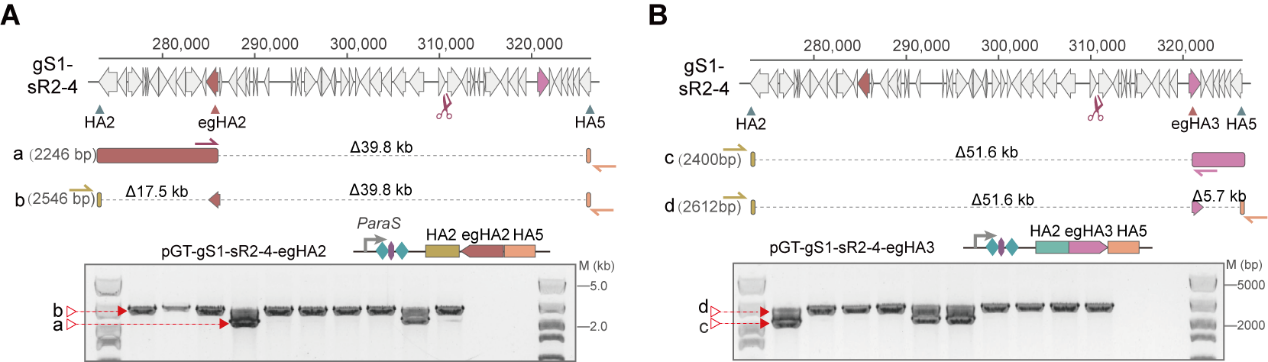


**Supplementary Figure S5.** **Replacing the gS1-sR2-4 segments with RT containing essential gene homology arms**

The genomic editing results after introducing the *SiRe_0319* (egHA2) **(A)** or *SiRe_0355* (egHA3) **(B)** gene into the multi-homology arm arrays of plasmids pGT-gS1-sR2-4-eHA2 and pGT-gS1-sR2-4-eHA3, respectively. Dotted lines delineate the region of deletion.


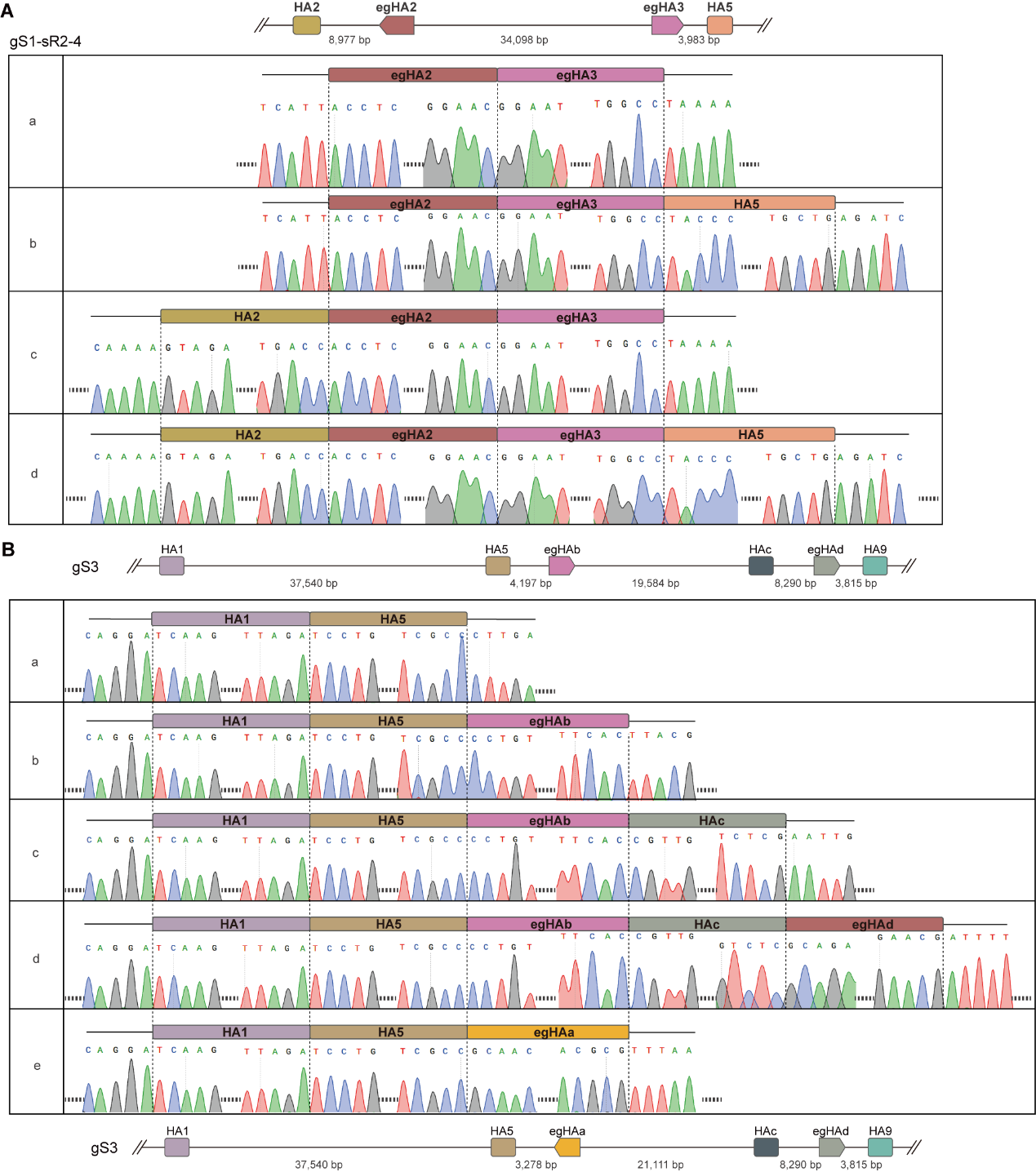


**Supplementary Figure S6. Sanger sequencing validation of transformants generated by CREAT**

Sanger sequencing results of each PCR product band after gel extraction from the PCR genotyping of gS1-Sr2-4 **(A)** and gS3 **(B)** transformants in Figure 3. The dashed black line indicates the unshown sequence results. The schematic diagram illustrates the positions of each homologous arm within the wild-type strain and the corresponding sizes of individual sub-regions.


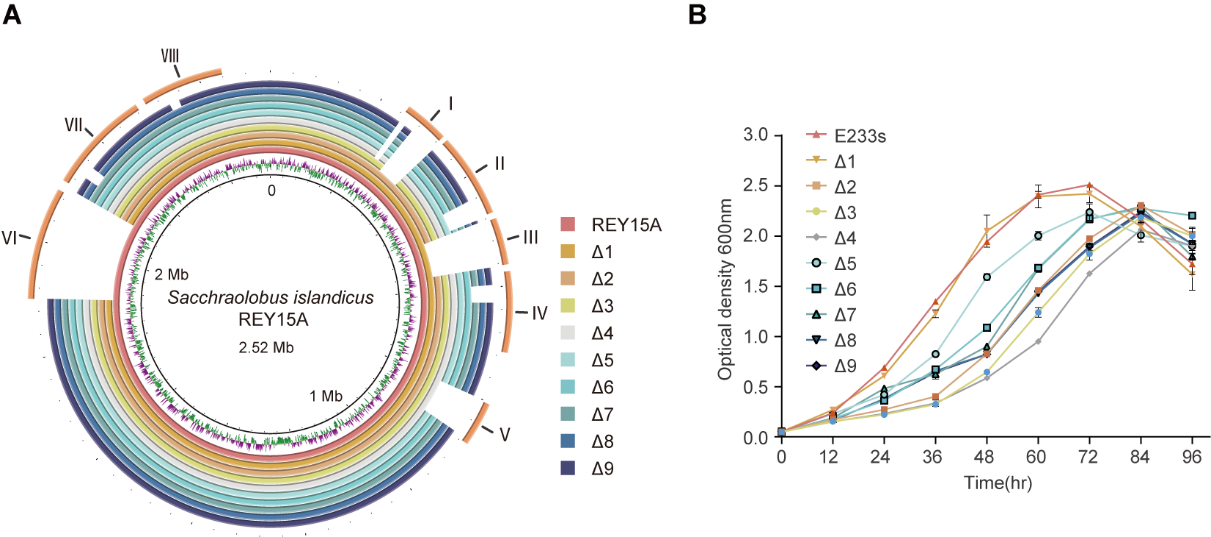


**Supplementary Figure S7.** **Genome trimming with CREAT strategy in *Sa. islandicus* REY15A.**

**A.** The CREAT strategy was systematically applied to eight deletable genomic loci, which were marked by the outermost orange arcs (Roman numerals Ⅰ-Ⅷ), and then a genome-wide comparative visualization map was generated. Nine iterative deletion mutants (numbered Δ1 to Δ9 in sequence) were successfully constructed, represented by concentric circles from the innermost to the outermost. Blank areas indicate the deleted regions. REY15A represents the wild-type strain. **B.** The growth profiles of genome-reduction mutants. Data are presented as mean values ± SD. n=3 biologically independent samples.


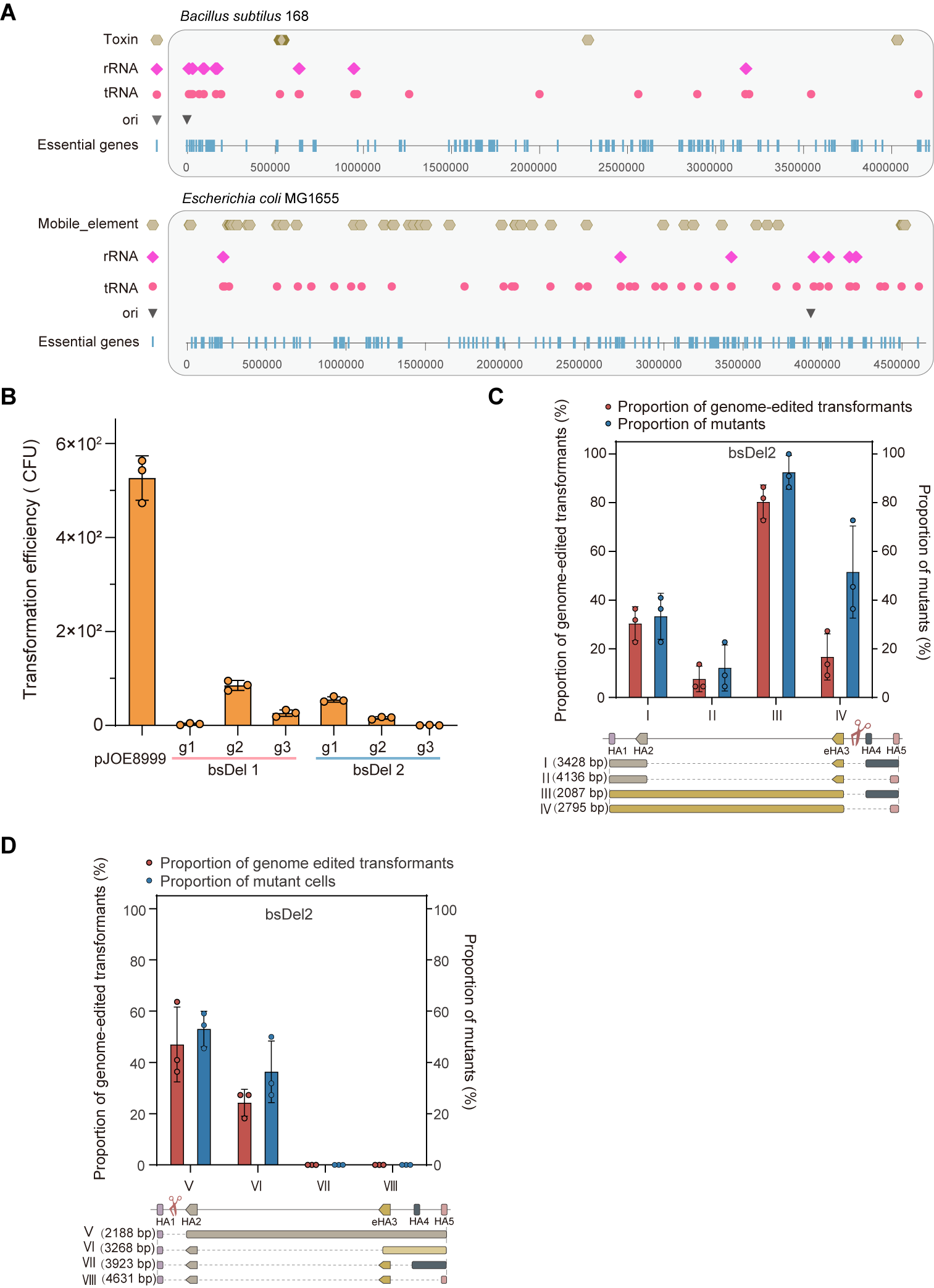


**Supplementary Figure S8. Genome-trimming with CREAT strategy in *Bacillus subtilis* 168**

**A.** Essential gene maps of *B. subtilis* strain 168 and *E. coli* MG1655. Known essential genes, rRNA and tRNA genes, mobile genetic elements and replication origins (*ori*) of *B. subtilis* 168 ^2^ and *E. coli* MG1655 ^3^ are highlighted with the symbols shown on the left panel of the figure. **B.** Colony formation units after transforming cells with genome-targeting plasmids. Three gRNAs targeting distinct positions were designed within both bsDel1 and bsDel2 regions, respectively. Values are means of three biological replicates each. **C.** Genome editing result with Cas9-based CREAT using a pGT-bsDel2-g3 plasmid targeting the bsDel2 region. Roman numerals Ⅰ-Ⅳ denote genotypes of different mutant strains. Values are means of three biological replicates. **D.** Genome editing result with Cas9-based CREAT using the pGT-bsDel2-g1 plasmid targeting the bsDel2 region. The CRISPR target site is located between the first and second homology arms. Dotted lines delineate the region of deletion. Values are means of three biological replicates each.


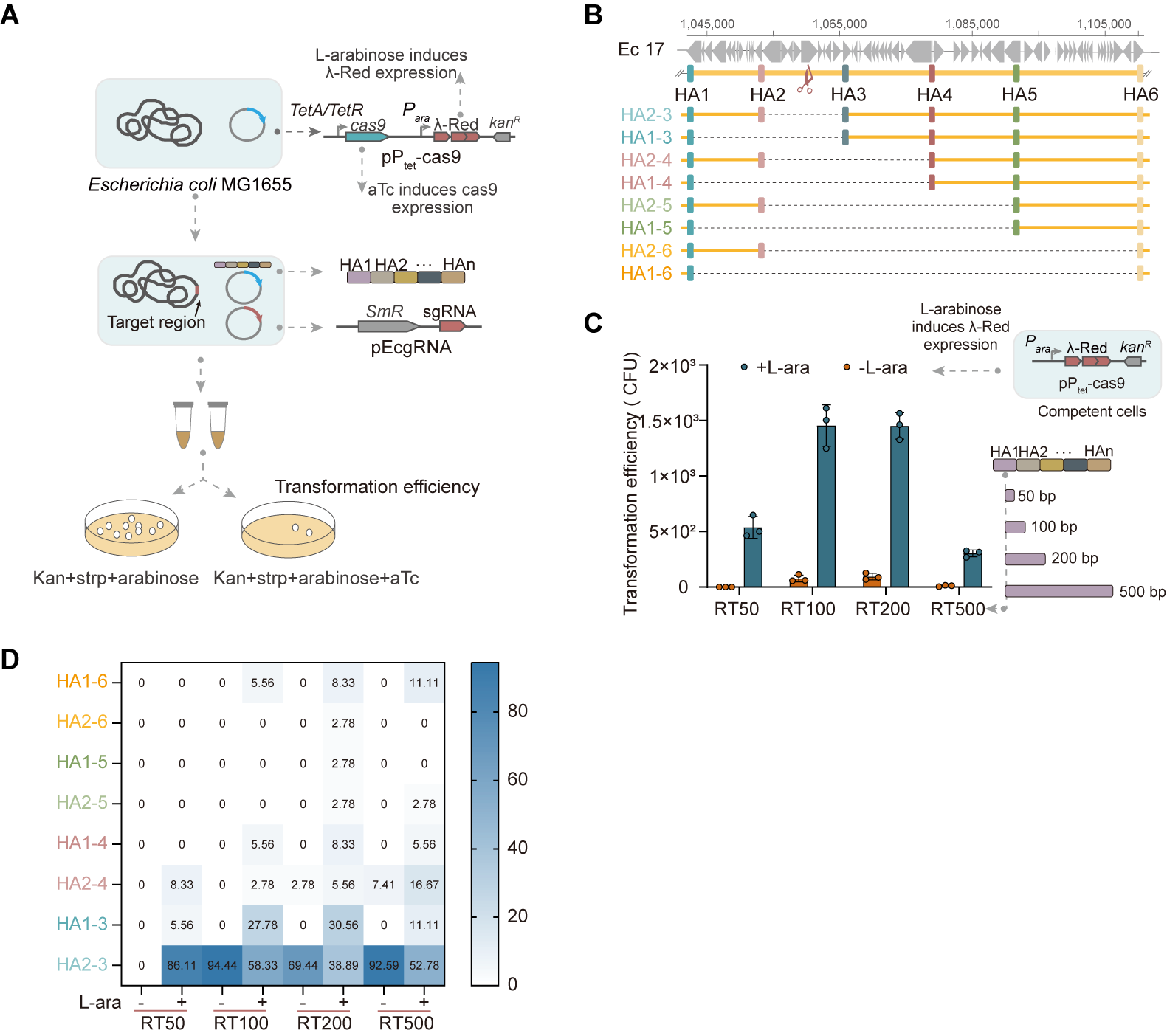


**Supplementary Figure S9. Combining CREAT strategy and λ-Red system for large fragment deletion in *E. coli***

**A.** Schematic diagram of non-essential region screening in *E. coli* MG1655 using the λ-Red system in conjunction with the CREAT method. The λ-Red system is induced by L-arabinose, while the Cas9 system is induced by anhydrotetracycline. **B.** Location of different homology arms (HA1-HA6) in the Ec17 region (1,041,971-1,109,876) of *E. coli* MG1655 genome. The schematic diagram shows genotypes of all theoretical recombinants in Ec17. **C.** Impacts of the length of homology arms and induction of the λ-Red system on the transformation efficiency. RT50, RT100, RT200 and RT500 refer to homologous arms of 50 bp, 100 bp, 200 bp and 500 bp, respectively. The bar graph shows the transformation efficiency of the pGT plasmid with/without the expression of λ-Red system. The expression of Cas9 protein was induced during the preparation of competent cells. Values are means of three biological replicates. **D.** The heatmap shows the frequency of different recombination events under each condition. Darker color indicates a higher frequency of occurrence. Values are means of three biological replicates.


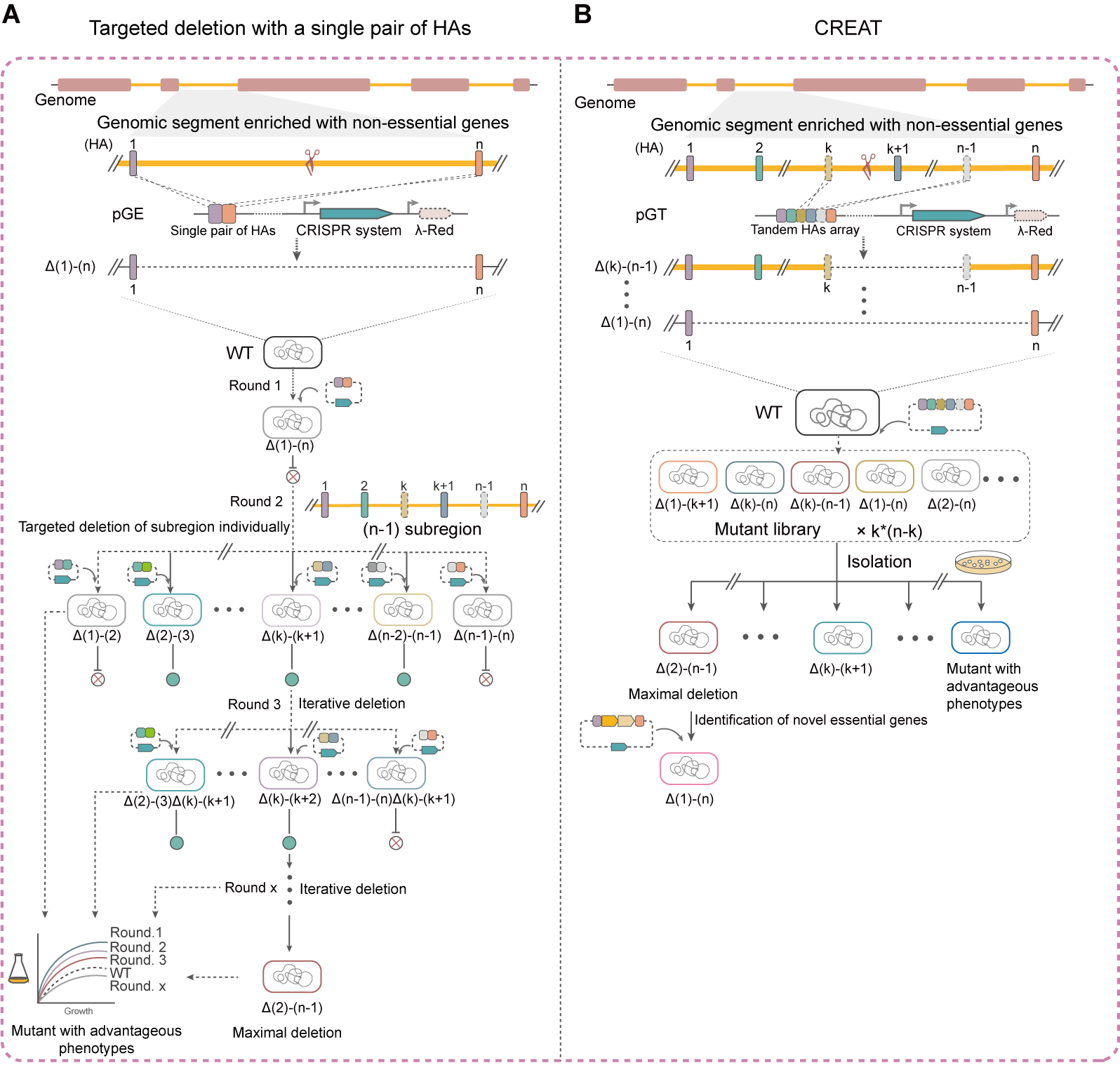


**Supplementary Figure S10. A summary of the CREAT strategy in recognizing large dispensable region and screening mutants of advantageous growth rate**

**A.** A workflow of targeted deletion with a single pair of HAs based on classical CRISPR genome editing. Since the presence of putative essential segments, the whole target region is arbitrarily divided into n-1 sub-regions, for which each targeted deletion experiment is conducted individually. This will reveal the essentiality of each segment/subregion, and for those deletable ones, iterative combinational deletion experiments are conducted until a mutant with the maximal deletion is achieved. Finally, by monitoring the growth phenotypes of all obtained mutants, mutants with advantageous phenotypes can be screened out. The green circles indicate the successfully obtained mutants. **B.** Workflow of the CREAT strategy. Multiple homologous arms (HA1, ..., HAn) are designed to divide the target genomic segment into n-1 subregions, with one target site located at HA(k)-(k+1) subregion (or any other subregions). These arms are fused to create a tandem multi-HA array (HA1-HAn) and cloned into the genome-targeting plasmid, which serves as a repair template following targeted genome cleavage induced by the CRISPR system. Recombination between the pGT plasmid and two different HAs flanking the target site generates a mutant library comprising n*(n-k) mutants. Subsequent plate-based phenotypic screening enables the isolation of mutants exhibiting either deletion or advantageous phenotypes.

**Table S1 Functional annotations of genes in the gS1 region**

| Region | Gene | Location | Length | Product |
| --- | --- | --- | --- | --- |
| gS1-sR1 | SiRe_0281 | 247672..248127 | 151 | methylated DNA-protein cysteine methyltransferase |
|  | SiRe_0283 | 249499..250254 | 251 | IS200/IS605 family OrfB protein |
|  | SiRe_0284 | 250342..251301 | 319 | IS5 family transposase |
|  | SiRe_0285 | 252025..252816 | 263 | sulfocyanin |
|  | SiRe_0286 | 252856..253620 | 254 | Hypothetical protein |
|  | SiRe_0287 | 253664..254647 | 327 | iron-containing alcohol dehydrogenase |
|  | SiRe_0288 | 254649..255095 | 148 | Hypothetical protein |
|  | SiRe_0289 | 255230..255835 | 201 | Hypothetical protein |
|  | SiRe_0290 | 255967..256884 | 305 | Alpha/beta hydrolase fold-3 domain-containing protein |
|  | SiRe_0291 | 256962..257972 | 336 | alcohol dehydrogenase GroES domain-containing protein |
|  | SiRe_0292 | 257958..259145 | 395 | acetyl-CoA acetyltransferase |
|  | SiRe_0293 | 259183..260082 | 299 | acyl-CoA dehydrogenase |
|  | SiRe_0294 | 260087..261013 | 308 | ribonucleotide reductase |
|  | SiRe_0295 | 261103..261861 | 252 | short-chain dehydrogenase/reductase SDR |
|  | SiRe_0296 | 261896..262942 | 348 | alcohol dehydrogenase GroES domain-containing protein |
|  | SiRe_0297 | 263226..263630 | 134 | Hypothetical protein |
|  | SiRe_0298 | 263694..265085 | 463 | Vinylacetyl-CoA Delta-isomerase |
|  | SiRe_0299 | 265495..265632 | 45 | Hypothetical protein |
|  | SiRe_0300 | 265878..267365 | 495 | major facilitator superfamily protein |
|  | SiRe_0301 | 267605..268204 | 199 | TetR family transcriptional regulator |
|  | SiRe_0302 | 268206..268649 | 147 | Fis family transcriptional regulator |
|  | SiRe_0303 | 268740..269882 | 380 | acetyl-CoA C-acetyltransferase (acetoacetyl-CoAthiolase) (AcaB-6) |
|  | SiRe_0304 | 269879..270235 | 118 | Hypothetical protein |
|  | SiRe_0305 | 270303..271973 | 556 | AMP-dependent synthetase and ligase |
|  | SiRe_0306 | 271997..273109 | 370 | acyl-CoA dehydrogenase |
|  | SiRe_0307 | 273135..275126 | 663 | 3-hydroxyacyl-CoA dehydrogenase NAD-binding protein |
| gS1-sR2 | SiRe_0308 | 275208..276140 | 310 | beta-lactamase |
|  | SiRe_0309 | 276349..277815 | 488 | major facilitator superfamily protein |
|  | SiRe_0310 | 277923..278237 | 104 | esterase |
|  | SiRe_0311 | 278227..278346 | 39 | Hypothetical protein |
|  | SiRe_0312 | 278435..278602 | 55 | Hypothetical protein |
|  | SiRe_0313 | 278690..279604 | 304 | beta-lactamase |
|  | SiRe_0314 | 279649..280602 | 317 | fumarylacetoacetate (FAA) hydrolase |
|  | SiRe_0315 | 280596..281525 | 309 | Alpha/beta hydrolase fold-3 domain-containing protein |
|  | SiRe_0316 | 281559..282503 | 314 | Aryldialkylphosphatase |
|  | SiRe_0317 | 282583..284265 | 560 | AMP-dependent synthetase and ligase |
|  | SiRe_0318 | 284321..284764 | 147 | carbon monoxide dehydrogenase subunit G |
|  | SiRe_0319 | 284768..286066 | 432 | FAD dependent oxidoreductase |
|  | SiRe_0320 | 286267..286497 | 76 | glycerol kinase |
|  | SiRe_0321 | 287220..288092 | 290 | ABC transporter |
|  | SiRe_0322 | 288089..289087 | 332 | daunorubicin resistance ABC transporter ATP-binding subunit, DrrA |
|  | SiRe_0323 | 289080..289487 | 135 | PadR family transcriptional regulator |
|  | SiRe_0324 | 289889..290116 | 75 | Hypothetical protein |
|  | SiRe_0325 | 290180..290347 | 55 | Hypothetical protein |
|  | SiRe_0326 | 291083..291577 | 164 | thioesterase superfamily protein |
|  | SiRe_0327 | 294000..294146 | 48 | Hypothetical protein |
|  | SiRe_0328 | 294452..294712 | 86 | Hypothetical protein |
|  | SiRe_0329 | 294689..295090 | 133 | Hypothetical protein |
|  | SiRe_0330 | 295435..296658 | 407 | NAD(FAD)-dependent dehydrogenase |
| gS1-sR3 | SiRe_0331 | 296827..297219 | 130 | Hypothetical protein |
|  | SiRe_0332 | 297336..298301 | 321 | cellulase (endo 1,4 beta glucanase), putative (CelB) |
|  | SiRe_0333 | 298290..298703 | 137 | Hypothetical protein |
|  | SiRe_0334 | 298745..298882 | 45 | Hypothetical protein |
|  | SiRe_0335 | 299373..300167 | 264 | transglutaminase domain-containing protein |
|  | SiRe_0336 | 300298..300744 | 148 | Hypothetical protein |
|  | SiRe_0337 | 300982..301446 | 154 | AsnC family transcriptional regulator |
|  | SiRe_0338 | 301497..302513 | 338 | thiamine pyrophosphate domain-containing TPP-binding protein |
|  | SiRe_0339 | 302532..303779 | 415 | pyruvate flavodoxin/ferredoxin oxidoreductase, alpha subunit |
|  | SiRe_0340 | 303776..304867 | 363 | pyruvate ferredoxin, flavodoxin oxidoreductase, delta subunit |
|  | SiRe_0341 | 305077..306369 | 430 | Oxalate/Formate Antiporter |
|  | SiRe_0342 | 306777..308369 | 530 | L-lactate transport |
|  | SiRe_0344 | 309981..310685 | 234 | Hypothetical protein |
|  | SiRe_0345 | 310928..312442 | 504 | amidase |
|  | SiRe_0346 | 312464..313111 | 215 | peroxiredoxin family protein |
| gS1-sR4 | SiRe_0347 | 313396..313761 | 121 | dinitrogenase iron-molybdenum cofactor biosynthesis protein |
|  | SiRe_0348 | 313758..313919 | 53 | Hypothetical protein |
|  | SiRe_0349 | 314217..314405 | 62 | Hypothetical protein |
|  | SiRe_0350 | 314868..315059 | 63 | Hypothetical protein |
|  | SiRe_0351 | 315064..316644 | 526 | Na+/solute symporter |
|  | SiRe_0352 | 316685..317494 | 269 | amidohydrolase 2 |
|  | SiRe_0353 | 317621..318592 | 323 | Hypothetical protein |
|  | SiRe_0354 | 318641..319450 | 269 | Hypothetical protein |
|  | SiRe_0355 | 320690..321979 | 429 | FAD dependent oxidoreductase |
|  | SiRe_0356 | 321982..322497 | 171 | Hypothetical protein |
|  | SiRe_0357 | 322509..323318 | 269 | amidohydrolase 2 |
|  | SiRe_0358 | 323346..323981 | 211 | Hypothetical protein |
|  | SiRe_0359 | 323962..324579 | 205 | thymidylate kinase-like protein |
|  | SiRe_0360 | 324563..325204 | 213 | dTMP kinase |

**Table S2 Distribution of FAD-type oxidoreductases in the genome of *Sa. islandicus* REY15A**

| Gene | Annotation | Description |
| --- | --- | --- |
| SiRe_0050 | Glycine/D-amino acid oxidase (deaminatng) | Essential gene |
| SIRe_0145 | Geranylgeranyl reductase, flavoprotein | Overlap with essential gene |
| SiRe_0211 | Q-Phytoene dehydrogenase or related enzyme | Overlap with essential gene |
| SiRe_0319 | Glycerol-3-phosphate dehydrogenase | N1-R2 |
| SiRe_0355 | Q-Phytoene dehydrogenase or related enzyme | N1-R4 |
| SiRe_1101 | Glycine/D-amino acid oxidase (deaminatng) | Near essential gene |
| SiRe_1752 | R-Uncharacterized FAD-dependent dehydrogenase | Near essential gene |
| SiRe_1973 | Glycine/D-amino acid oxidase (deaminatng) | Near essential gene |
| SiRe_2400 | I-Geranylgeranyl reductase, flavoprotein | Near essential gene |
| SiRe_2459 | I-Geranylgeranyl reductase, flavoprotein | Essential gene |

**Table S3 functional annotations of genes in the gS3 region**

| **Region** | **Gene** | **Location** | **Length** | **Product** |
| --- | --- | --- | --- | --- |
| **gS3-sR1** | SiRe_0836 | 800536..801324 | 262 | family 2 glycosyl transferase |
|  | SiRe_0837 | 802082..803275 | 397 | Hypothetical protein |
|  | SiRe_0838 | 803262..803879 | 205 | Hypothetical protein |
|  | SiRe_0839 | 804207..804374 | 55 | Hypothetical protein |
|  | SiRe_0840 | 804547..805488 | 313 | dTDP-D-glucose 4,6-dehydratase |
|  | SiRe_0841 | 805485..806525 | 346 | glucose-1-phosphate thymidylyltransferase |
|  | SiRe_0842 | 806526..807350 | 274 | dTDP-4-dehydrorhamnose reductase |
|  | SiRe_0843 | 807704..808240 | 178 | dTDP-4-dehydrorhamnose 3,5-epimerase |
|  | SiRe_0844 | 808340..809059 | 239 | type 11 methyltransferase |
|  | SiRe_0845 | 809185..809502 | 105 | Hypothetical protein |
|  | SiRe_0846 | 809644..809916 | 90 | Hypothetical protein |
|  | SiRe_0847 | 809918..810370 | 150 | Hypothetical protein |
|  | SiRe_0848 | 810478..811218 | 246 | Hypothetical protein |
|  | SiRe_0849 | 811557..811778 | 73 | Hypothetical protein |
|  | SiRe_0850 | 811853..812890 | 345 | family 2 glycosyl transferase |
| **gS3-sR2** | SiRe_0851 | 813225..813356 | 43 | Hypothetical protein |
|  | SiRe_0852 | 813725..814408 | 227 | IS6 family transposase |
|  | SiRe_0853 | 814783..815691 | 302 | FkbM family methyltransferase |
|  | SiRe_0854 | 816777..817886 | 369 | group 1 glycosyl transferase |
|  | SiRe_0855 | 818131..818313 | 60 | Hypothetical protein |
|  | SiRe_0856 | 818509..819342 | 277 | IS110 family transposase |
| **gS3-sR3** | SiRe_0857 | 819682..820632 | 316 | IS110 family transposase |
|  | SiRe_0858 | 820886..821215 | 109 | IS6 family transposase |
|  | SiRe_0859 | 821181..821492 | 103 | Hypothetical protein |
|  | SiRe_0861 | 822816..823712 | 298 | family 2 glycosyl transferase |
|  | SiRe_0862 | 823843..824238 | 131 | Hypothetical protein |
|  | SiRe_0863 | 824256..824618 | 120 | Hypothetical protein |
|  | SiRe_0864 | 824888..825328 | 146 | Hypothetical protein |
|  | SiRe_0865 | 825346..825783 | 145 | Hypothetical protein |
|  | SiRe_0866 | 825900..826508 | 202 | Hypothetical protein |
|  | SiRe_0867 | 827173..828207 | 344 | glycosyl transferase family 1 |
|  | SiRe_0868 | 828219..829130 | 303 | family 2 glycosyl transferase |
|  | SiRe_0869 | 829169..831472 | 767 | Hypothetical protein |
| **gS3-sR4** | SiRe_0870 | 831807..831926 | 39 | Hypothetical protein |
|  | SiRe_0872 | 834102..834245 | 47 | Hypothetical protein |
|  | SiRe_0873 | 834508..834693 | 61 | Hypothetical protein |
|  | SiRe_0874 | 835055..835816 | 253 | family 2 glycosyl transferase |
|  | SiRe_0875 | 835948..837249 | 433 | sulfatase-like protein |
|  | SiRe_0876 | 838085..839548 | 487 | Hypothetical protein |
| **gS3-sR5** | SiRe_0877 | 839572..840243 | 223 | Hypothetical protein |
|  | SiRe_0878 | 840379..841365 | 328 | FkbM family methyltransferase |
|  | SiRe_0879 | 841365..842270 | 301 | phosphoadenosine phosphosulfate reductase |
|  | SiRe_0880 | 842563..843984 | 473 | polysaccharide biosynthesis protein |
|  | SiRe_0881 | 844100..844819 | 239 | FkbM family methyltransferase |
|  | SiRe_0882 | 845351..845518 | 55 | Hypothetical protein |
|  | SiRe_0883 | 845545..846507 | 320 | ATPase (AAA+ superfamily) |
|  | SiRe_0884 | 847576..848940 | 454 | CRISPR-associated (Cas) DxTHG family |
| **gS3-sR6** | SiRe_0885 | 849044..850249 | 401 | IS200/IS605 family OrfB protein |
|  | SiRe_0886 | 850224..850631 | 135 | IS200/IS605 family OrfA transposase |
|  | SiRe_0888 | 851954..852184 | 76 | VapB-type antitoxin |
|  | SiRe_0889 | 852430..852630 | 66 | VapC-type toxin |
|  | SiRe_0890 | 852841..853623 | 260 | Cmr4 family CRISPR-associated RAMP protein |
|  | SiRe_0891 | 853620..854090 | 156 | CRISPR-associated protein, Cmr5 |
|  | SiRe_0892 | 854083..855486 | 467 | Cmr1 family CRISPR-associated RAMP protein |
|  | SiRe_0893 | 855486..856223 | 245 | Cmr6 family CRISPR-associated RAMP protein |
|  | SiRe_0894 | 856208..858856 | 882 | CRISPR-associated protein, Cmr2 family |
| **gS3-sR7** | SiRe_0895 | 858863..859681 | 272 | CRISPR-assciated protein, Cmr3 |
|  | SiRe_0897 | 860733..861920 | 395 | DEXX-box ATPase-like protein |
|  | SiRe_0899 | 863654..864178 | 174 | archaeal PaREP1/PaREP8 family |
|  | SiRe_0900 | 864450..864947 | 165 | ISH3 family transposase |
|  | SiRe_0901 | 865024..865983 | 319 | IS5 family transposase |
|  | SiRe_0902 | 866328..866714 | 128 | HEPN domain-containing protein |
|  | SiRe_0903 | 866690..867034 | 114 | Hypothetical protein |
|  | SiRe_0904 | 867367..867888 | 173 | PaREP1 family protein |
|  | SiRe_0905 | 868151..869244 | 364 | ISNCY family transposase |
| **gS3-sR8** | SiRe_0906 | 869728..870216 | 162 | Hypothetical protein |
|  | SiRe_0907 | 870278..871414 | 378 | Fe-S oxidoreductase |
|  | SiRe_0908 | 871387..872439 | 350 | Fe-S oxidoreductase family protein |
|  | SiRe_0909 | 872565..873974 | 469 | aldehyde dehydrogenase |
|  | SiRe_0910 | 874273..874431 | 52 | Hypothetical protein |
|  | SiRe_0911 | 874877..875119 | 80 | Hypothetical protein |
|  | SiRe_0912 | 875620..876711 | 363 | archaeal ATPase (AAA+ superfamily) |
|  | SiRe_0913 | 877100..878164 | 354 | group 1 glycosyl transferase |

**Table S4** The detailed information of iterative generation of multiple genomic deletions

| **Mutants** | **Deletion regions(start-end)** | **Deletion size** | **Genome size** |
| --- | --- | --- | --- |
| WT | —— | —— | 2,522,992 (100%) |
|  |  |  |  |
| Δ1 | 1,901,926-2,101,133 | 199.20 kb | 2,323,765 (92.10%) |
|  |  |  |  |
| Δ2 | 481,619-564,744 | 83.13 kb | 2,240,640 (88.81%) |
|  |  |  |  |
| Δ3 | 275,564-325,068 | 50.39 kb | 2,190,245 (86.81%) |
|  |  |  |  |
| Δ4 | 247,687-259,960 | 12.27 kb | 2,177,972 (86.32%) |
|  |  |  |  |
| Δ5 | 799,829-872,413 | 72.58 kb | 2,105,388 (83.45%) |
|  |  |  |  |
| Δ6 | 438,773-476,773 | 38.00 kb | 2,067,388 (81.94%) |
|  |  |  |  |
| Δ7 | 595,825-628,087 | 32.26 kb | 2,035,126 (80.66%) |
|  |  |  |  |
| Δ8 | 2,132,411-2,154,503 | 22.09 kb | 2,103,034 (83.35%) |
|  |  |  |  |
| Δ9 | 2,335,223-2,353,804 | 18.58 kb | 1,994,453 (79.05%) |
|  |  |  |  |

**References**

1. Zhang, C., Phillips, A.P.R., Wipfler, R.L., Olsen, G.J. & Whitaker, R.J. The essential genome of the crenarchaeal model Sulfolobus islandicus. *Nat Commun* **9**, 4908 (2018).

2. K. Kobayashi et al. Essential Bacillus subtilis genes. *Proc. Natl. Acad. Sci. USA* **100**, 4678-4683 (2003).

3. Baba, T. et al. Construction of Escherichia coli K‐12 in‐frame, single‐gene knockout mutants: the Keio collection. *Mol. Syst. Biol.* **2** (2006).
