# Supplementary material for: CREAT: A CRISPR‐Based Genome Trimming Strategy for Systematic Identification of Dispensable Regions and Rapid Genome Reduction
Source: Adv Sci (Weinh). 2026 Jun 29:e76042. Online ahead of print. doi: 10.1002/advs.76042 (PMC13337030; doi:10.1002/advs.76042)

Source Data- Figure 2b

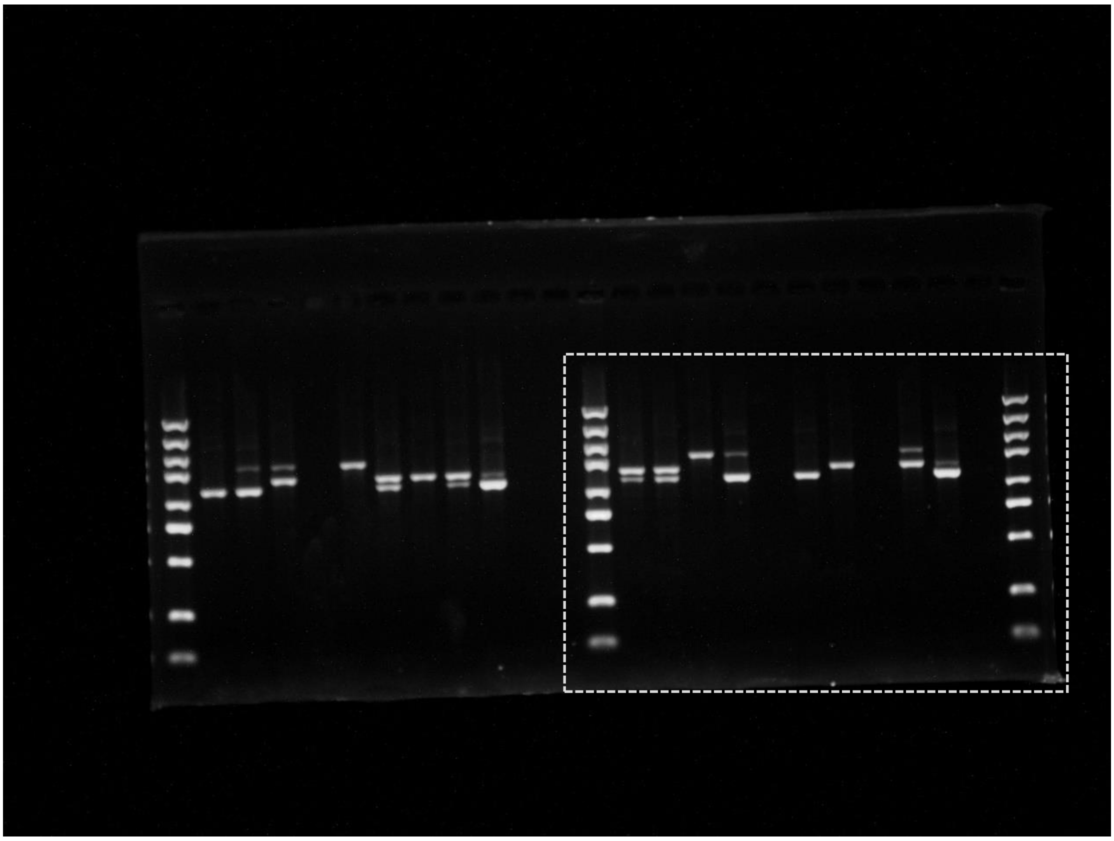

Source Data- Figure 2c

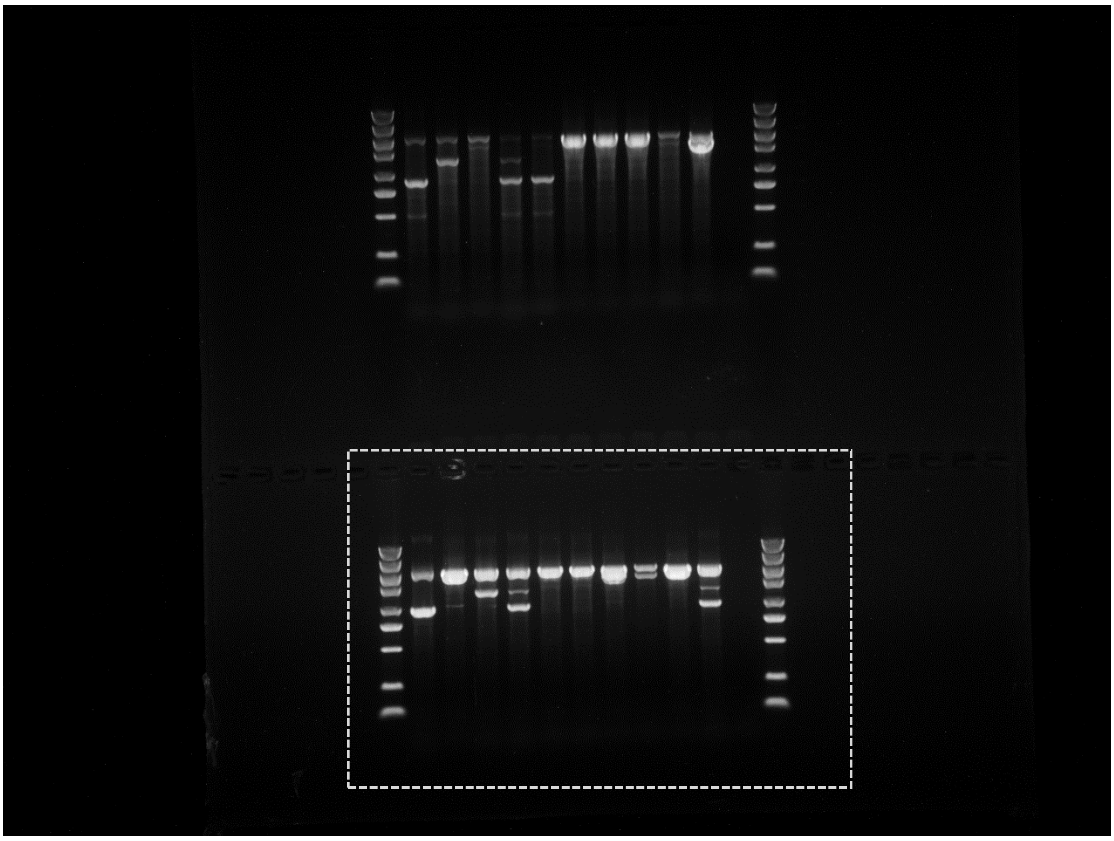

Source Data- Figure 2d

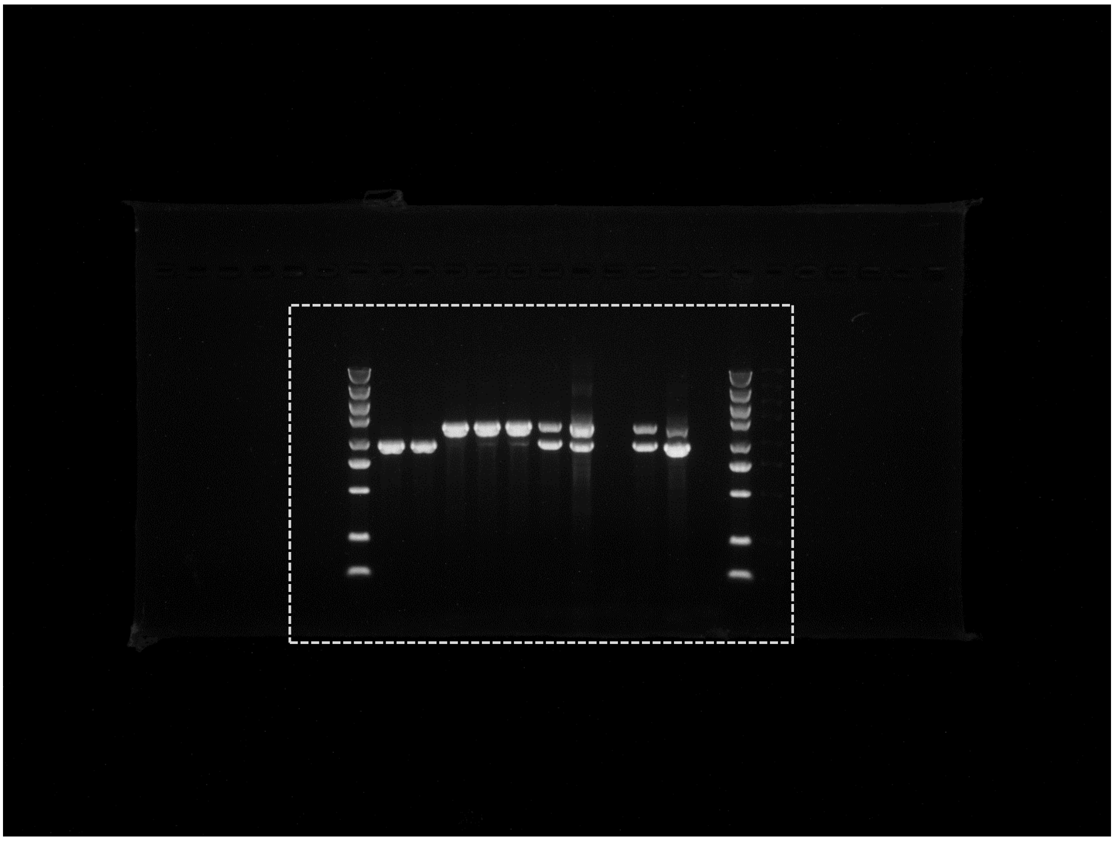

Source Data- Figure 3c

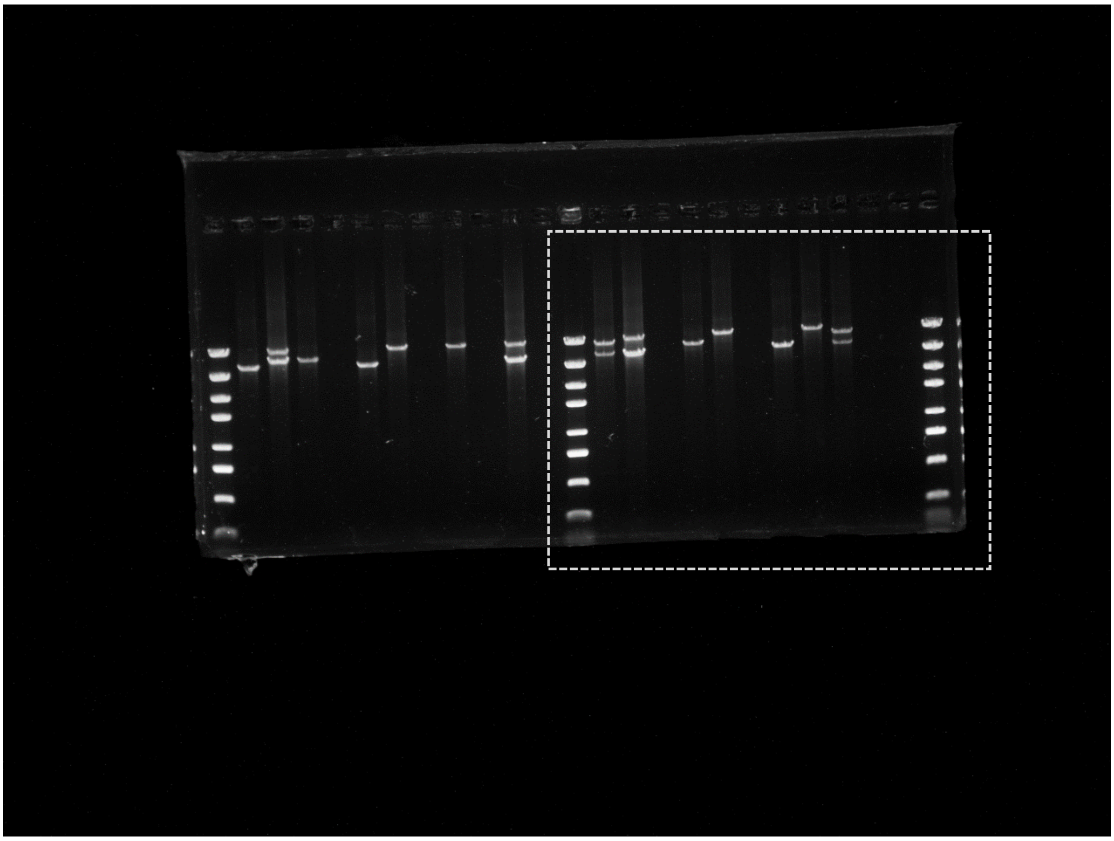

Source Data- Figure 3e (pGeTha-gS3-eHAb)

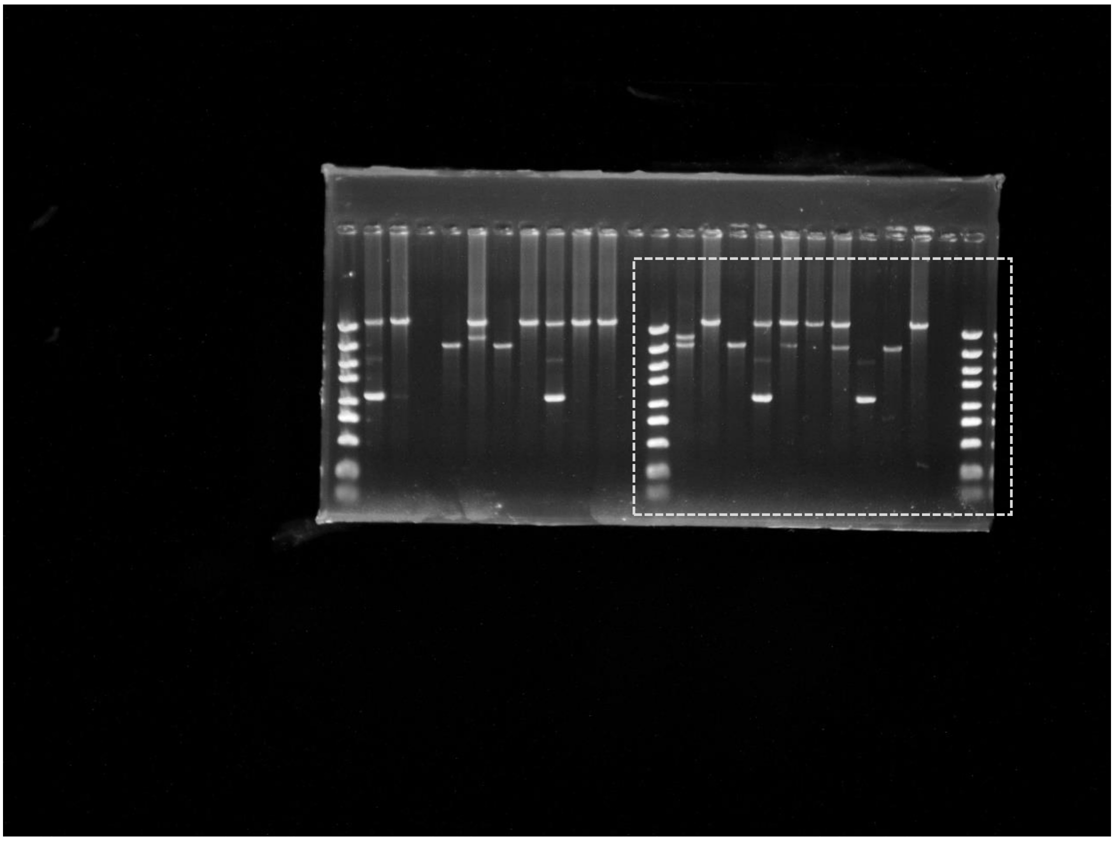

Source Data- Figure 3e (pGeTha-gS3-eHAa)

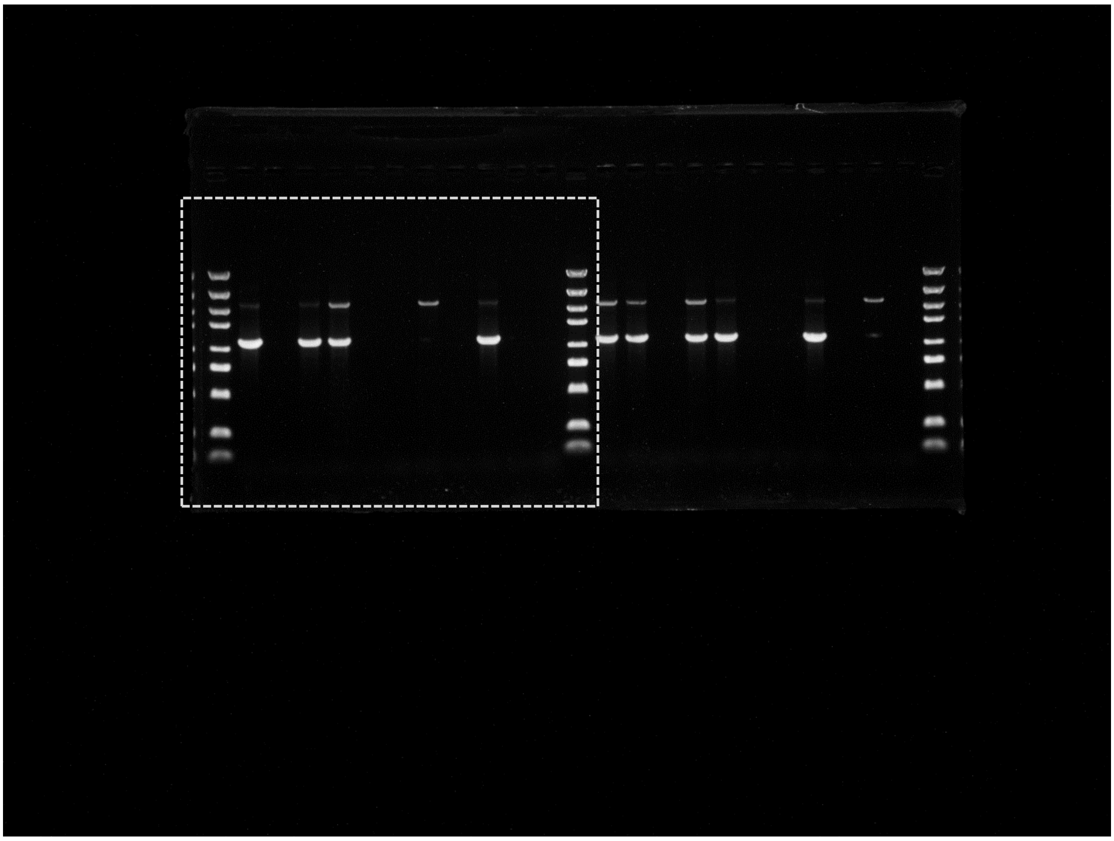

Supplement: Supplementary file 2 — Supporting File 2: advs76042‐sup‐0002‐DataSet.zip. [file ADVS-9999-e76042-s001.zip › Source Data-gel image.pdf]
